# Androgen receptor as a mediator and biomarker of radioresistance in triple-negative breast cancer

**DOI:** 10.1038/s41523-017-0038-2

**Published:** 2017-08-18

**Authors:** Corey Speers, Shuang G. Zhao, Ben Chandler, Meilan Liu, Kari Wilder-Romans, Eric Olsen, Shyam Nyati, Cassandra Ritter, Prasanna G. Alluri, Vishal Kothari, Daniel F. Hayes, Theodore S. Lawrence, Daniel E. Spratt, Daniel R. Wahl, Lori J. Pierce, Felix Y. Feng

**Affiliations:** 1 Department of Radiation Oncology, University of Michigan, Ann Arbor, MI USA; 2 Breast Oncology Program, University of Michigan Comprehensive Cancer Center, Ann Arbor, MI USA; 3 Comprehensive Cancer Center, University of Michigan, Ann Arbor, MI USA; 4 University of California San Francisco, San Francisco, CA USA

## Abstract

Increased rates of locoregional recurrence have been observed in triple-negative breast cancer despite chemotherapy and radiation therapy. Thus, approaches that combine therapies for radiosensitization in triple-negative breast cancer are critically needed. We characterized the radiation therapy response of 21 breast cancer cell lines and paired this radiation response data with high-throughput drug screen data to identify androgen receptor as a top target for radiosensitization. Our radiosensitizer screen nominated bicalutamide as the drug most effective in treating radiation therapy-resistant breast cancer cell lines. We subsequently evaluated the expression of androgen receptor in >2100 human breast tumor samples and 51 breast cancer cell lines and found significant heterogeneity in androgen receptor expression with enrichment at the protein and RNA level in triple-negative breast cancer. There was a strong correlation between androgen receptor RNA and protein expression across all breast cancer subtypes (*R* ^2^ = 0.72, *p* < 0.01). In patients with triple-negative breast cancer, expression of androgen receptor above the median was associated with increased risk of locoregional recurrence after radiation therapy (hazard ratio for locoregional recurrence 2.9–3.2)) in two independent data sets, but there was no difference in locoregional recurrence in triple-negative breast cancer patients not treated with radiation therapy when stratified by androgen receptor expression. In multivariable analysis, androgen receptor expression was most significantly associated with worse local recurrence-free survival after radiation therapy (hazard ratio of 3.58) suggesting that androgen receptor expression may be a biomarker of radiation response in triple-negative breast cancer. Inhibition of androgen receptor with MDV3100 (enzalutamide) induced radiation sensitivity (enhancement ratios of 1.22–1.60) in androgen receptor-positive triple-negative breast cancer lines, but did not affect androgen receptor-negative triple-negative breast cancer or estrogen-receptor-positive, androgen receptor-negative breast cancer cell lines. androgen receptor inhibition with MDV3100 significantly radiosensitized triple-negative breast cancer xenografts in mouse models and markedly delayed tumor doubling/tripling time and tumor weight. Radiosensitization was at least partially dependent on impaired dsDNA break repair mediated by DNA protein kinase catalytic subunit. Our results implicate androgen receptor as a mediator of radioresistance in breast cancer and identify androgen receptor inhibition as a potentially effective strategy for the treatment of androgen receptor-positive radioresistant tumors.

## Radiation: Drug target found for radioresistance

Drugs that block the androgen receptor (AR) protein can help make radiation-resistant breast tumors susceptible to ionizing therapy. Corey Speers from the University of Michigan, Ann Arbor, USA, and colleagues characterized the radiation sensitivity of 21 breast cancer cell lines and then paired the response data with the findings of a high-throughput drug screen to identify a medication called bicalutamide — an AR inhibitor — as one of the most potent agents for overcoming radiation resistance. The researchers then measured the expression of AR in more than 2100 human breast tumor samples and 51 breast cancer cell lines, and found that patients with triple-negative breast cancer, especially those who relapsed after radiation, had elevated levels of AR. Cell experiments with enzalutamide, a newer generation AR-blocking drug, confirmed that targeting AR helps reverse radiation resistance.

## Introduction

Radiation therapy (RT), in addition to surgery and systemic therapy, remains a mainstay of current clinical management of breast cancer. Although effective in most women, some will develop recurrent disease despite multi-modality therapy, including a significant percentage of the ~42,000 women diagnosed with triple-negative breast cancer (TNBC) each year.^1^ Studies detailing the poor response of TNBC to adjuvant RT underscore the biologic differences and as yet undefined oncogenic drivers of these particular types of tumors, with TNBC much less likely to have significant local- and disease-free survival advantages from adjuvant RT and chemotherapy treatment in women.^2–4^ Given the lack of targeted agents for TNBC and their relative RT insensitivity (as evidenced by their increased locoregional recurrence risk) the development of additional targets for radiosensitization represents a critical unmet clinical need.

Recent genomic profiling studies have identified a significant subgroup of TNBC that express androgen receptor (AR) and are susceptible to androgen receptor blockade.^5^ This finding suggests that at least some patients with TNBC may respond to treatment with androgen receptor blockade and provides a potentially effective, molecular strategy for women diagnosed with TNBC. Indeed, multiple clinical trials are assessing the effect of androgen receptor blockade in patients with metastatic breast cancer whose tumors express AR (NCT03055312, NCT01889238, NCT02580448, NCT01889238, NCT00468715, NCT00755885- clinicaltrials.gov).

While these studies aim to determine the clinical utility of treating women with TNBC who have metastatic disease, recent data by our group and others suggests that AR-blockade may be an effective radiosensitization strategy in the upfront, definitive setting.^6, 7^ In this study we employed a novel radiosensitizing screen to identify potential targets for radiosensitization. This screen, which coupled the RT response of 21 breast cancer cell (BCC) lines using clonogenic survival assays with high-throughput drug screen data, identified AR inhibition as a top target for radiosensitization. Herein, we report significant heterogeneity in AR RNA and protein expression levels in human breast cancer, including TNBC. We demonstrate significant correlation of AR RNA and protein expression levels across all subtypes of breast cancer. Furthermore, we demonstrate that in clinical data sets of women treated with breast-conserving surgery and radiation, AR expression level is a potential predictive biomarker of local recurrence and radiation response in women with TNBC. We demonstrate that second generation antiandrogens confer radiosensitivity in AR-positive, TNBC cell lines as well as in vivo in xenograft models. Bioinformatic approaches nominate DNA repair efficiency as a possible mechanism of AR-mediated radioresistance, and subsequent mechanistic studies demonstrate that AR expression is induced by ionizing radiation. Finally, we show that intact AR function is associated with effective resolution of double stranded DNA breaks induced by ionizing radiation, and inhibition of AR function significantly delays this repair at least in part through a DNA protein kinase catalytic subunit (DNAPKcs)-mediated mechanism.

## Results

**Fig. 1:**
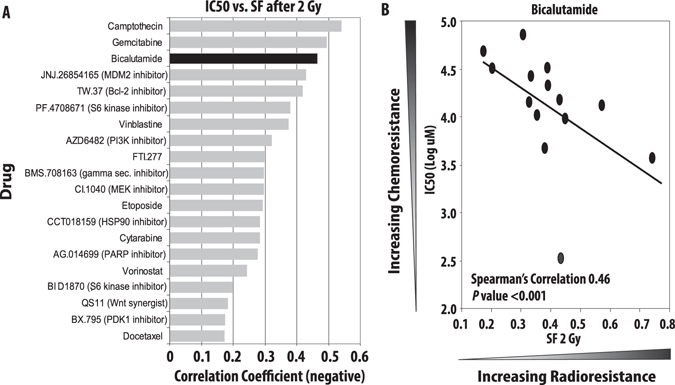
A novel radiosensitizer screen nominates AR-inhibition as one of the most effective strategies in treating radioresistant BCC lines. Clonogenic survival assays were performed to determine the intrinsic RT sensitivity of 21 BCC lines (0–8 Gy RT) with significant heterogeneity in intrinsic radiosensitivity of the BC cell lines. IC50 values were determined for 130 clinically available compounds and correlation coefficients were calculated using IC50 values (for drug sensitivity) and surviving fraction after 2 Gy of radiation (SF-2Gy for radiation sensitivity). Bicalutamide was identified as one of the most effective drugs for treatment of radiation-resistant breast cancers (**a**) with a correlation coefficient of 0.46 (**b**)

In an effort to identify more effective therapies for breast cancers with intrinsic radioresistance, we designed a novel “drug radiosensitivity” screen which combined the intrinsic radiosensitivity information obtained from clonogenic survival data with drug sensitivity values (IC50 values) from 130 characterized, clinically available drugs using the Genomics of Drug Sensitivity in Cancer (GDSC) database.^8^ Our previous work determined the intrinsic radiosensitivity of a panel of 21 breast cancer cell lines and identified heterogeneity in radiation sensitivity that was breast cancer intrinsic subtype independent (Supplementary Table 1).^9^ In this study, we sought to identify clinically available drugs that might be effective in treating breast cancers with intrinsic radioresistance. We therefore coupled the intrinsic radiosensitivity data, as measured by the surviving fraction of cells after 2 Gy of radiation (SF-2 Gy), with the IC50 values of all drugs in breast cancer cell lines in the GDSC database (97 drugs; Supplementary Table 2). Our radiosensitizer screen nominated bicalutamide as one of the most effective drugs in treating radioresistant BCC lines, with only camptothecin and gemcitabine, two well characterized and established radiosensitizers, being more strongly correlated in our screen (Fig. 1). Given previous studies documenting AR expression in TNBC and the known radioresistance of this particular type of breast cancer to radiation, we sought to further explore the relationship of AR expression to radiation sensitivity.^5, 10^

**Fig. 2:**
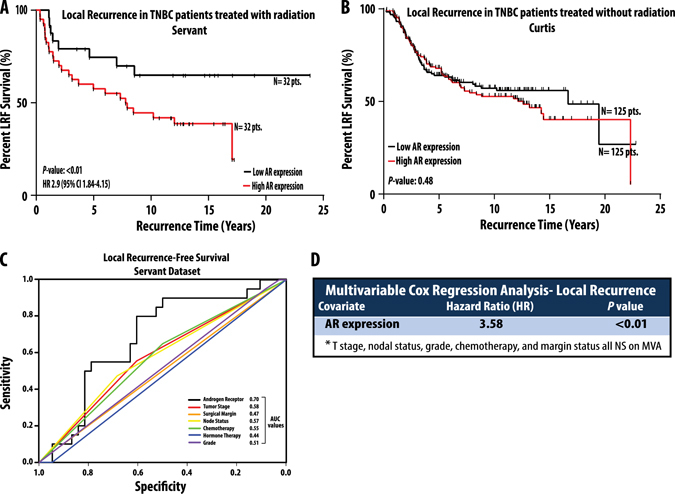
AR expression is predictive of response to radiation. Kaplan–Meier local recurrence-free survival analysis in the Servant data set demonstrates that patients with TNBC whose tumors have higher than median expression of AR (*red line*) have significantly higher rates of local recurrence after radiation and an overall poorer prognosis than patients with lower than median expression (*black line*) of AR (**a**). In patients with TNBC who did not receive radiation treatment, there was no difference in local recurrence depending on higher (*red line*) or lower (*black line*) than median expression of AR (**b**). Receiver operator curve analysis in the Servant data set demonstrates that AR expression level with the highest AUC value (0.69) when compared against other clinicopathologic parameters (**c**). In multivariable Cox regression analysis in the Servant data set, only AR expression remained significantly associated with worse local recurrence-free (*LRF*) survival (**d**). Hazards ratios, 95% confidence intervals, and *p*-values were calculated for all analyses and are listed

We began by interrogating the expression of AR in >2000 human breast tumor samples at both the RNA and protein level using the TCGA data set. This analysis demonstrated significant heterogeneity in AR expression in both TNBC and non-TNBC breast cancers (Supplementary Fig. 1A, B). In addition, there was a strong correlation between AR RNA and protein expression levels, and this correlation remained significant across all subtypes of breast cancer (Supplementary Fig. 1C). Given the noted expression of AR in TNBC, we sought to explore AR as a radiosensitizing strategy in the radioresistant TNBC cell lines and whether AR expression might serve as a predictive biomarker of response to ionizing radiation in women with TNBC. To that end we identified a human breast tumor data set for which gene expression levels were known and for which there was long-term follow-up, including local recurrence information available.^11^ The first data set (Servant et al.) included 343 patients with a minimum of 10-year follow-up and long-term locoregional recurrence events captured. Most of these patients had early stage node-negative disease managed with breast-conserving surgery, treated adjuvantly with radiation, and without adjuvant chemotherapy (Supplementary Table 3 for details of the cohort). Analysis was restricted to patients with TNBC (*N* = 64) and Kaplan–Meier recurrence-free survival (RFS) analysis showed that patients whose tumors had higher than median expression of AR had markedly higher rates of local recurrence after radiation with a hazard ratio (HR) for local recurrence 2.9 and a *p* < 0.01 (Fig. 2a **)**. To confirm these findings, we identified a second, independent data set with 69 TNBC patients that was similar in patient characteristics to the Servant data set (Supplementary Table 3 for details of the cohort).^12^ In this data set (van de Vijver) Kaplan–Meier RFS-analysis again showed that patients whose tumors had higher than median expression of AR had dramatically higher rates of local recurrence after radiation with a HR for local recurrence 2.8 and a *p* < 0.01. To determine whether AR expression level was not merely a prognostic biomarker of improved locoregional control but truly a predictive biomarker of response, we analyzed a publically available data set that included TNBC patients not treated with adjuvant radiation.^13^ This data set (Curtis), which included over 2000 breast cancer patients, contained details about radiation treatment and local recurrence and only those TNBC patients not treated with radiation were included in the analysis. In this case, there was no difference in local recurrence in TNBC patients (*N* = 150) not treated with radiation when stratified by AR expression (Fig. 2b), suggesting that AR expression was predictive of response to radiation, not merely prognostic of outcome independent of the treatment received.

**Fig. 3:**
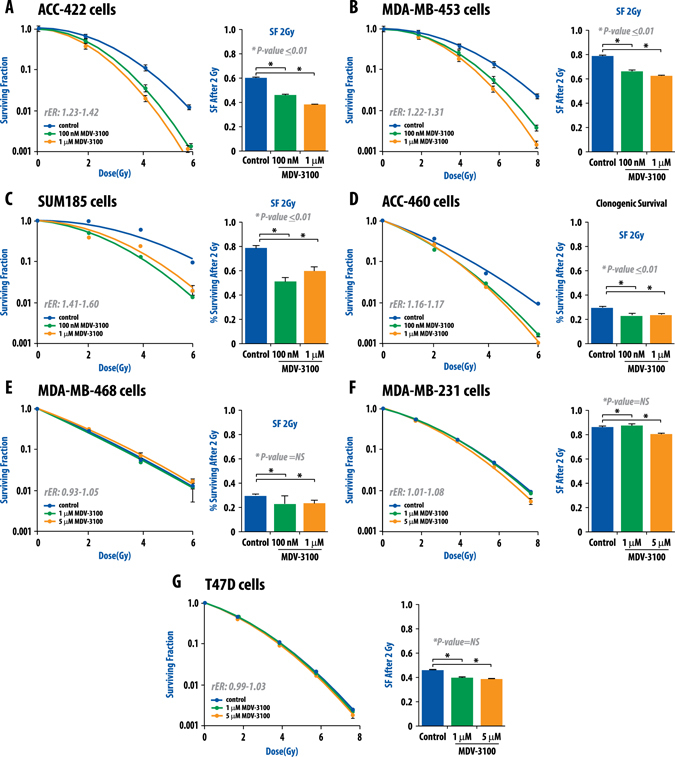
Clonogenic survival assays demonstrate that MDV3100 is an effective radiosensitizer in TNBC cell lines that have high expression of AR. Four cell lines (MDA-MB-453, ACC-422, SUM-185PE, and ACC-460) from the recently described luminal androgen receptor (*LAR*) subtype of TNBC were selected and treated with varying doses of ionizing radiation and MDV3100. Clonogenic survival assays were performed and both surviving fraction after 2 Gy and enhancement ratios were calculated (**a**–**d**). Treatment with MDV3100 effectively radiosensitized the LAR cell lines (**a**–**d**). MDA-MB-468 (**e**) and MDA-MB-231 (**f**) TNBC cell lines with low AR expression were not significantly radiosensitized by MDV3100, nor was the AR-negative, ER-positive cell line T47D (**g**). Radiation enhancement ratios (*rER*) and surviving fraction after 2-Gy (SF-2Gy) values are depicted. Experiments were repeated at least in triplicate and *error bars* represent SEM

In the data set of patients treated with radiation (Servant), receive operator curve (ROC) analysis to assess sensitivity and specificity of AR as a predictor of local recurrence identified AR as most predictive of local recurrence in patients treated with RT with an AUC of 0.70 (Fig. 2c). Subsequent univariate analysis of factors associated with local control in TNBC demonstrated AR expression level as most significantly associated with local recurrence after radiation treatment. Furthermore, multivariable analysis of all clinical and pathological factors previously shown to be associated with local control after radiation (T-stage, nodal status, grade, chemotherapy, and margin status) showed AR expression level (analyzed as a continuous variable) was most significantly associated with local recurrence with a HR of 3.58; *p* < 0.01 (Fig. 2d). Thus, AR was associated with decreased local control rates only in irradiated patients. This observation suggests that low AR-expression predicts for a favorable response to radiation and inhibition of AR signaling in AR overexpressing TNBC could improve local control in this population. To further explore this possibility, we designed a series of experiments to examine the effect of antiandrogen treatment on the radiation sensitivity of TNBC cell lines with varying expression levels of AR. We first determined the expression levels of AR in various breast cancer cell lines (RNA and protein) and found significant heterogeneity in AR expression levels in breast cancer cell lines (Supplementary Fig. 2A, B). As MDA-MB-453 and ACC-422 were TNBC cell lines with the highest AR expression levels, they were chosen for further investigation. Inhibition of AR using the AR signaling inhibitor and antagonist MDV3100 (enzalutamide) significantly radiosensitized the AR+TNBC cell lines in a dose dependent manner, with radiation enhancement ratios (rER) ranging from 1.22 to 1.42 (Fig. 3a, b). This level of radiosensitization is comparable to the level of the radiosensitization achieved with the well-known radiosensitizer cisplatin (rER 1.2). Two additional AR + TNBC cell lines (SUM-185PE and ACC-460) with slightly lower AR expression from the previously described luminal AR subtype of TNBC^5^ also showed significant radiosensitization with MDV3100 treatment with rER of 1.16–1.60 (Fig. 3c, d). No such radiosensitization was seen in the AR-negative TNBC cell lines MDA-MB-231, MDA-MB-468, or the AR-negative, estrogen-receptor (ER)-positive breast cancer cell line T47D (Fig. 3e–g).

**Fig. 4:**
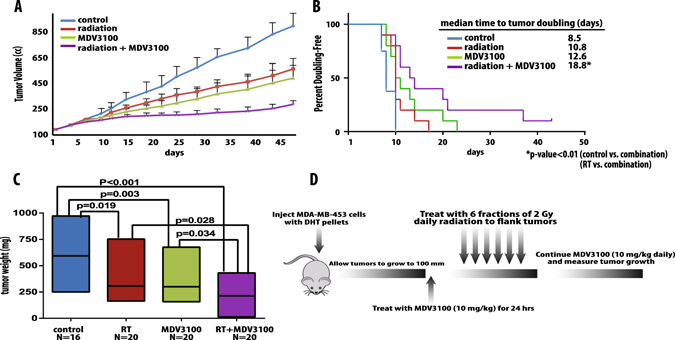
AR inhibition through with MDV3100 significantly radiosensitized TNBC xenografts with AR expression in mouse models and markedly delayed tumor doubling time and tumor weight. CB17-SCID mice were injected with 1 × 10^6^ MDA-MB-453 cells and tumors were allowed to grow to ~100 mm^3^. Treatment was then initiated as depicted in the four treatment groups (control, RT alone, MDV3100 alone, and RT + MDV3100). Tumor volume and weight were tracked, and time to tumor doubling was plotted (**a**–**c**). A schema of treatment is included (**d**)

Having demonstrated that AR inhibition radiosensitizes multiple AR-positive breast cancer cell lines, we then evaluated the effect of AR inhibition in vivo. For these xenograft studies, MDA-MB-453 AR-positive TNBC cells were injected into the bilateral flanks of SCID mice. These mice were treated with the AR inhibitor MDV3100 daily via oral gavage after tumors reached a volume of ~100 mm^3^, with MDV3100 treatment initiated 24 h before radiation administration. Although inhibition of AR using MDV3100 and radiation treatment each independently inhibited tumor volume growth in these models, the combination of MDV3100 and RT treatment resulted in a significant (*p* < 0.01) and synergistic decrease in tumor growth compared to RT alone. Furthermore, when time to tumor doubling time was calculated, there was an almost doubling of the time with combination treatment compared to RT treatment alone (Fig. 4a, b **)**. Additionally, there was a marked decrease in the tumor weight in the combination treated group when compared to all other treatment groups (Fig. 4c). On target effects of the drug were confirmed by harvesting the treated xenograft tumors and checking AR and AR target gene expression by quantitative radiation therapy PCR (qRT-PCR) (Supplementary Fig. 3). A schematic of the treatment schema is depicted in Fig. 4d. Treatment with MDV3100 in combination with RT did not result in significant toxicity in the mice with no discernable difference in growth, weight, or fur between the treated and untreated mice. This toxicity profile is consistent with reports from other groups.^14^

**Fig. 5:**
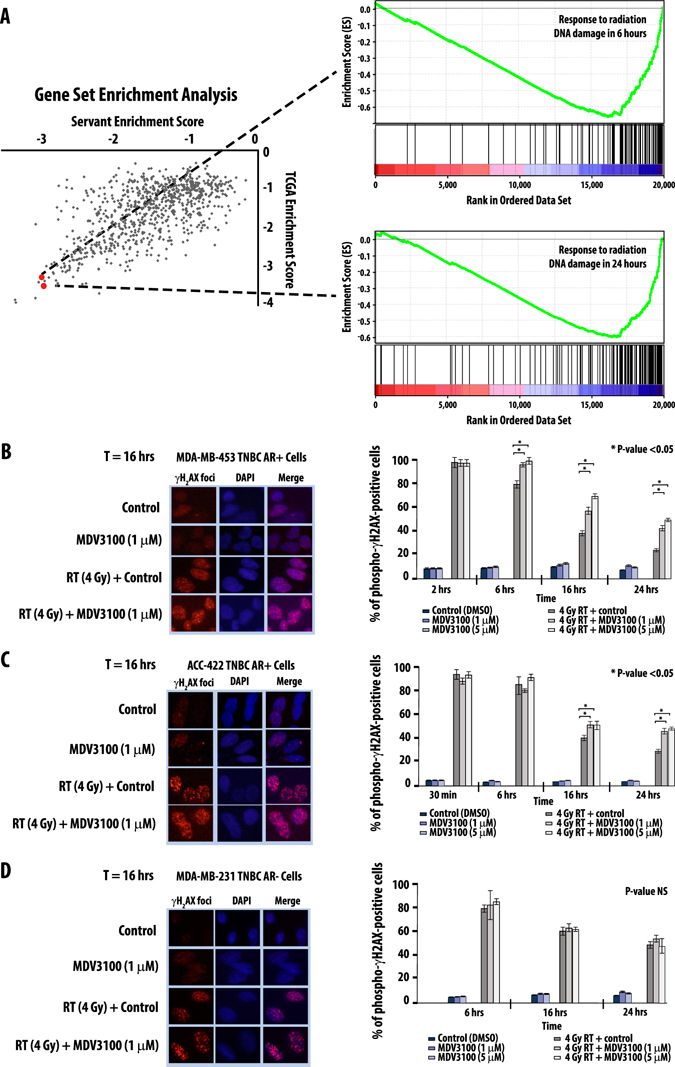
GSEA analysis identifies DNA repair after ionizing radiation as a top concept associated with AR expression (**a**). AR inhibition through with MDV3100 significantly delays double stranded DNA break repair at 2, 6, and 16 h in the AR-positive TNBC cell lines MDA-MB-453 (**b**) and ACC-422 (**c**) but not the AR-negative TNBC cell line MDA-MB-231 (**d**). Cells were treated with MDV3100, RT, or combination and γH2AX foci were manually counted. Images are representative of cells are the indicated time points. Minimum of 100 cells per condition were counted, and experiments were repeated in triplicate. *Error bars* represent SD

While our studies identified AR as being implicated in radioresistance in vitro and in vivo, we wanted to further explore the underlying mechanisms of AR-induced radioresistance in TNBC. We first evaluated whether AR expression was induced by ionizing radiation. AR-positive breast cancer cell lines were treated with 4 Gy of ionizing radiation and AR RNA and protein levels were evaluated at 1, 12, 24, and 48 h after radiation treatment. AR RNA levels were significantly increased after radiation treatment, but there was not a concomitant increase in AR protein levels (Supplementary Fig. 4A, B). Despite the increase in AR RNA levels, the moderate levels of induction and lack of protein expression increases suggested that AR-mediated radioresistance was likely not exclusively mediated by a mere upregulation of AR expression. To explore additional mechanisms of AR-mediated radioresistance we performed gene set enrichment analysis (GSEA) to identify concepts associated with AR expression across all published gene expression data sets. AR gene expression was correlated to every sequenced gene in the TN samples in both the TCGA and Servant data sets. The ranked gene lists were then imputed into GSEA analysis as previously described.^15^ GSEA analysis identified gene sets related to radiation induced DNA damage response were several of the top negatively-associated concepts in both data sets (Fig. 5a).

Having identified DNA damage repair as significantly overrepresented concept in GSEA analysis, we interrogated the role of AR in DNA damage repair. As ionizing radiation confers lethality through the introduction of double stranded DNA (dsDNA) breaks, we sought to assess what, if any, role AR played in the timing and efficiency of double stranded DNA damage repair using gamma H2AX foci formation assays. Not surprisingly, AR inhibition with MDV3100 alone did not significantly increase γH2AX foci in the AR + TNBC line MDA-MB-453 at 2, 6, 16, or 24 h, suggesting that MDV3100 treatment alone was not inducing dsDNA breaks (Fig. 5b). As expected, there was an increase in dsDNA breaks after 4 Gy of ionizing radiation at those same time points, but these breaks were almost completely resolved by 24 h in MDA-MB-453 cells treated with radiation alone (Fig. 5b). Combination treatment with 1 μM MDV3100 and 4 Gy radiation, however, resulted in a significant delay of dsDNA break repair at 6, 16, and 24 h (Fig. 5b). To confirm that this was not an isolated cell line phenomenon, confirmatory experiments in the AR-positive TNBC cell line ACC-422 showed similar results (Fig. 5c). No such delay in dsDNA break repair was seen in γH2AX assays in the AR-negative, ER-negative MDA-MB-231 cell line (Fig. 5d) or the AR-negative, ER-positive T47D cell lines (data not shown). Thus AR inhibition with the antiandrogen MDV3100 significantly decreased the degree and rate of dsDNA break repair after ionizing radiation, suggesting that AR function played a critical role in the repair and resolution of dsDNA breaks.

**Fig. 6:**
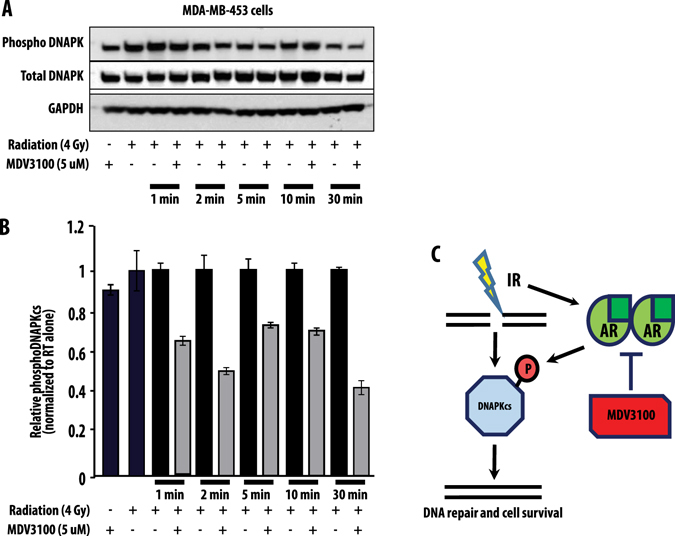
Total DNAPKcs levels are unchanged by RT treatment, but treatment with MDV3100 decreased phosphorylated DNAPKcs levels after ionizing radiation. Total DNAPKcs and phosphoDNAPKcs levels were measured ±RT and ±MDV3100 treatment at 1, 2, 5, 10, and 30 min (**a**). Quantification of phosphoDNAPKcs changes ±MDV3100 radiation treatment are depicted (**b**). Mechanistically, AR functions to allow for active phosphorylation of DNAPKcs after leading to more efficient resolution of dsDNA breaks induced by ionizing radiation. This activation is impaired by antiandrogen therapy (**c**). Experiments were repeated in triplicate and *error bars* represent SD

While these experiments demonstrated that AR functioned in the repair of dsDNA breaks, the mechanism remained unclear. Previous studies from our group and others identified DNAPKcs as a mediator of AR-inducted radioresistance in prostate cancer, and we hypothesized that a similar mechanism may be acting in TNBC.^6, 16^ DNAPKcs is a serine/threonine kinase involved in non-homologous end joining, the key repair pathway responsible for repair of dsDNA breaks induced by ionizing radiation. To determine whether DNAPKcs was involved in AR-mediated radioresistance, we evaluated the expression level of DNAPKcs after ionizing radiation treatment. DNAPKcs protein levels were largely unchanged by ionizing radiation alone, suggesting that total increases in protein levels were likely not responsible for AR-mediated repairs of dsDNA breaks (Fig. 6a). Recognizing that DNAPKcs activation was dependent upon phosphorylation at Ser2056 to be active, we next interrogated the functional activation of DNAPKcs by evaluating the expression changes of phospho-DNAPKs (pDNAPKcs) with and without radiation treatment. In AR + TNBC cell lines, treatment with 4 Gy of radiation led to increase in pDNAPKcs expression levels (Fig. 6a), suggesting that ionizing RT was sufficient to activate DNAPKcs. To further investigate the role of AR inhibition on pDNAPKcs levels after radiation, we first evaluated MDV3100 treatment alone on pDNAPKcs levels and found that MDV3100 treatment did not affect pDNAPKcs levels at multiple time points (Supplementary Fig. 5). We then compared expression levels of pDNAPKcs with and without MDV3100 treatment with radiation treatment (4 Gy) at early and late time points. As activation of DNAPKcs is known to be an immediate event after ionizing radiation, we found that AR + TNBC cell lines treated with MDV3100 had a significant decrease in pDNAPKcs levels at multiple time points within the first 30 min after radiation (Fig. 6b), suggesting AR function was necessary for early activation of DNAPKcs (by phosphorylation of Ser2065) and dsDNA break repair. These findings suggest that AR function is necessary for the early activation of the critical DNA repair protein DNAPKcs, and inhibition of AR with MDV3100 leads to early inhibition of DNAPKcs phosphorylation and subsequent activation.

## Discussion

In this study we performed a novel radiosensitizer screen that identified anti-androgen therapy as a potentially effective strategy for the treatment of AR-positive radioresistant breast cancers. RNA and protein expression profiling of over 2000 human breast tumors demonstrates that AR RNA and protein expression is strongly correlated across all subtypes of breast cancer, and that significant heterogeneity exists amongst all subtypes for AR expression, suggesting potential benefit outside of TNBC. In clinical data sets of women treated with breast-conserving surgery and radiation, AR expression level is a potential predictive biomarker of local recurrence and radiation response in women with TNBC treated with radiation. Inhibition of AR function using second generation antiandrogens confers radiosensitivity to traditionally radioresistant AR-positive, TNBC cell lines and this radiosensitization occurs across many AR-positive TNBC cell lines. Furthermore, in vivo studies show that this radiosensitization can be conferred by oral administration of MDV3100 in xenograft model systems. GSEA analysis identified response to radiation induced DNA damage as significantly associated with AR expression and AR inhibition significantly impaired resolution of dsDNA breaks. Mechanistically, AR inhibition impairs the efficiency and timing of dsDNA break repair which may be mediated, at least in part, through expression, phosphorylation, and activation of DNAPKcs. Taken collectively, these studies demonstrated that antiandrogen therapy may be an effective means of treating women with AR-positive, radioresistant breast cancers and provides the preclinical rationale for initiation of early phase clinical trials of combination antiandrogens and radiotherapy for women with AR-positive, triple-negative breast cancer at high risk of local recurrence.

This work builds upon a mounting body of literature exploring the role of targeted radiation sensitizing agents. Indeed, previous studies adding targeted therapies to radiation have demonstrated that the efficacy and synergistic effects of targeted therapies as radiosensitizers, including antiandrogens.^6, 17^ Furthermore, our group and others have shown that AR upregulation can indeed mediate radioresistance in prostate cancer,^18^ and this work extends those findings into breast cancer.^17^ Based on this preclinical data there are ongoing clinical trials assessing the efficacy radiation coupled with PARPi, gemcitabine, or lapatinib (NCT01868503) in breast cancer, mTOR inhibitors in prostate cancer (NCT01642732), Wee-1 inhibition (with MK-1775) in cervical cancer (NCT01958658), trametinib in rectal cancer (NCT01740648). These trials demonstrate not only the feasibility of combinatorial clinical trials, but the continued clinical interest in developing such treatment strategies. Combination therapy with MDV3100 and radiation in AR-positive breast tumors would extend this previous work beyond the general strategy of radiosensitization into the realm of molecularly and clinically targeted therapy.

Antiandrogen treatment as a radiosensitizing strategy offers a potentially attractive opportunity to improve outcomes in women with breast cancer. Multiple clinical trials using antiandrogens in the context of AR-positive metastatic breast cancer for women with both ER-positive and ER-negative breast tumors demonstrate not only a favorable toxicity profile in phase I studies, but subsequent encouraging response rates in the resulting phase II trials.^19–21^ Furthermore, data from the prostate cancer trials with second generation antiandrogens demonstrate very limited, if any, additional normal tissue toxicity of antiandrogen treatment in combination with radiation even when treating to much higher radiation doses than is customary for breast cancer treatment.^22, 23^ Furthermore, the recent demonstration that AR status does not change markedly through disease progression in breast cancer suggests that antiandrogen therapy may also be effective in early-stage disease, not just in the metastatic setting.^24^ Given the expression of AR in all subtypes of breast cancer described herein, not just in TNBC, suggests that antiandrogen treatment as a radiosensitizing strategy in AR-positive breast cancer may also be effective and is currently being evaluated further by our group. Indeed, if more generalizable, anti-androgen therapy may be a potentially effective treatment for all women with AR-positive breast cancer with limited added toxicity. In fact, although the luminal androgen receptor subtype of breast cancer was the focus of these studies, AR is known to be more highly expressed in ER-positive breast cancers, including luminal B subtype cancers, and may represent a novel and effective treatment opportunity in these patients. Additionally, whether there are differences in radiosensitization in the various subtypes of TNBC based on AR expression outside the luminal AR subtype remains unexplored. Given the effect on DNAPKcs mediated by AR inhibition, it is plausible that this strategy may be effective in the BL-1 subtype of TNBC which is known to be dependent on DNA repair pathways. While these unanswered questions remain an area of active interest within our group, these data add to a mounting body of clinical and preclinical data suggesting that antiandrogen therapies in combination with radiation may be an effective treatment strategy for women with treatment refractory and radiation-resistant breast cancer, including women with AR-positive triple-negative breast cancer.

## Methods

### Cell culture and cell lines

A variety of breast cancer cell lines were selected to intentionally recapitulate the variety of breast cancer subtypes. All lines were purchased from ATCC (Manassas, VA, USA) or DSMZ (Brunswick, Germany) between 2012 and 2015 as described previously.^9, 25, 26^ Cell lines were authenticated at the University of Michigan DNA Sequencing Core Facility with comparison to known sequences included from ATCC and DSMZ. All media, FBS and penicillin/streptomycin (50units/ml) were procured from Invitrogen, and all cell lines were maintained in a 5% CO2 cell culture incubator. The following breast cancer cell lines were grown in RPMI 1640 supplemented with 10% FBS: ZR75-30, MDA-MB-231, MDA-MB-453, BT474, BT20, AU565, HCC 1954, HCC 1806, HCC38, HCC70, ACC-231, and HCC 1937. ACC-302 cells were grown in 80% DMEM with 20% FBS. ACC-422 cells were grown in 85% MEM with 15% FBS. BT549 and T47D cells were grown in RPMI 1640 with 10% FBS and 0.023 IU/ml insulin. ACC-459, ACC-440, and CAMA-1 were grown in DMEM with 10% FBS. MCF-7 cells were grown in modified MEM with 0.023 IU/ml insulin in keeping with our previous studies.^25, 26^

### RNA isolation and quantitative RT-PCR: (Ben and Cassie)

RNA was isolated from cells or tissue using TRIzol (Invitrogen) extraction and miRNAeasy kit (Qiagen). The protocol included in the kit was followed, using all optional steps. RT was performed using RT Superscript III and random primers (Invitrogen). Primers, dNTPs, and RNA were first incubated at 65 °C for 5 min. Upon completion of the first incubation, salt buffers, RT SS III, and RNaseOUT were added and incubated at 25 °C for 5 min, 50 °C for 60 min, and 75 °C for 15 min. Upon completion of RT, cDNA was diluted 1:5 fold. A comparative qPCR was performed using SYBR Green (Applied Biosystems) and gene specific primers, ordered from Integrated DNA Technologies. The ΔΔCt values were calculated by first comparing genes of interest with a housekeeping gene (GAPDH) and subsequently comparing the condition of interest to the control condition for each gene to determine comparative gene expression. Data is represented as gene expression ±SEM.

### Western blot analysis

Western blot analysis was carried out using standard protocols as previously described.^15^ Breast cancer cells were grown in culture dishes and treated with select compounds or radiation for designated time periods. Cell lysates were made in RIPA buffer supplemented with protease and phosphatase inhibitors and were resolved on SDS–PAGE gels. The proteins were transferred to polyvinylidene difluoride membranes and probed using phospho-DNAPK (Abcam-CAT#124918), total DNAPK (Cell Signaling-CAT#12311), androgen receptor (Millipore-CAT#06-680) and GAPDH (Cell Signaling-CAT#2118L) antibodies followed by HRP-conjugated secondary antibodies (Sigma) then visualized using the enhanced chemiluminescence Western Blotting Detection Reagent. To isolate proteins from patient-derived tumors or mouse xenograft tumors, samples were homogenized in RIPA buffer and processed as above.

### Clonogenic survival assays

Clonogenic surivival assays performed as described previously.^26^ Briefly, exponentially growing cells in 6-well plates were treated with MDV3100, radiation, or both at concentrations noted in the figures. Treated cells were allowed to grow until distinct colonies were identified under ×4 magnification. They were then fixed and stained and colonies that consisted of ≥50 cells were counted as positive. As described previously,^15, 26^ plating efficiency was corrected for in all experiments and the effect of MDV3100 treatment (toxicity) was determined by comparing the number of alive treated cells relative to untreated cells. Cell survival curves were fitted using the linear-quadratic equation. The rER was calculated as the ratio of the mean inactivation dose under control conditions divided by the mean inactivation dose under drug treatment conditions as previously described.^15^

### Irradiation

All cell line and animal radiation treatment was done utilizing a Philips RT250 orthovoltage machine at a dose rate of ∼2 Gy/min in the University of Michigan Comprehensive Cancer Center Experimental Irradiation Core as described previously.^8^ Calibration was done utilizing a calibrated ionization chamber attached to an electrometer according to the National Institute of Standards and Technology calibration. For animal experiments mice received isofluorane for anesthetic and tumors were treated with tumors exposed and a 2.4 cm aperture utilized for collimation and the lead shields placed to cover the remainder of the normal tissue of each mouse.

### Mouse xenograft experiments

A total of 4 × 10^6^ MDA-MB-453 cells were subcutaneously injected bilaterally in the flank of 4–6-week-old female CB17-SCID mice. All mice were also implanted with 12.5 mg 60-day release 5-alpha-DHT pellets (Innovative Research of America, catalog #SA-161). After randomization to group by tumor size to ensure no differences at treatment outset, tumor growth and size was assessed three times each week for the duration of the experiment utilizing digital calipers with assessor blinded to treatment group. Average tumor volume was calculated using the following formula: (*π*/6) (*L* × *W*2), where *L*=tumor length and *W*=tumor width) as described previously.^15, 26^ When tumors reached ~100 mm^3^, mice were separated into four treatment groups and treatment was initiated as follows: vehicle control that received water via oral gavage once daily, MDV3100 dosed at 10 mg/kg via oral gavage once daily, RT (2 Gy for 6 days), and a combination RT (2 Gy for 6 days) plus MDV3100 (once daily at 10 mg/kg). In the combination group, mice were treated with MDV3100 for 24 h prior to the first dose of RT. Body weight was monitored weekly to assess weight loss during treatment. Experimental protocols and plan was reviewed and approved by, as well as monitored by, the Institutional Animal Care and Use Committee (IACUC). Tumor growth curve comparisons and statistical analysis was performed using unpaired *t*-test with two-tailed *p*-value for significance.

### Gamma H2AX foci formation

Analysis was performed as previously described.^15, 26^ Briefly, for cell staining and foci formation assays, cells were cultured on coverslips in 12-well plates and treated with MDV3100 as indicated for 24 h and then immediately exposed to 4 Gy radiation. Cells were collected at indicated time points (2, 6, 16, and 24 h) and processed. Images were collected with a ×60 objective lens. The γH2AX foci were detected with mouse monoclonal antibodies phosphor γH2AX (Millipore, Cat#05-636). For quantitation of γH2AX foci, at least 100 cells from each of three independent experiments were visually scored for each condition. Cells with ≥10 γH2AX foci were scored as positive and compared for statistical analyses.

### Patient cohorts

A publicly available clinical cohort with gene expression and local recurrence information was utilized for biomarker assessment (Servant) as previously described.^9^ As described previously, this multi-institutional cohort consisted of 343 patients from the Netherlands and France with early stage breast cancer treated with breast-conserving surgery with post-op radiation.^11^ An additional data set (van deVijver) was utilized as it represented a patient cohort with lymph-node-negative breast cancer treated with surgical resection as previously described.^12^ Clincopathologic characteristics of the cohorts are described in Supplementary Table 3. For prognostic analysis, a data set that included patients treated with various systemic therapies was utilized and only TNBC patient not treated with radiation were included in the analysis.^13^

All appropriate IRB protocols were followed in the acquisition and analysis of the data from these clinical data sets. Please refer to the original cited publications for full details of the IRB approval.

### Gene expression data

Normalized expression data for the cell lines was downloaded from the EMBL-EBI ArrayExpress website as described in the original publication^27^ and per our previous method described.^9^ Normalized expression data for the Servant data set was downloaded from the EMBL-EBI Array Express repository (http://www.ebi.ac.uk/arrayexpress/experiments/E-TABM-157/). All expression data was log transformed and median centered and scaled to the same minimum/maximum. Array expression data for the van de Vijver and Curtis data sets was obtained from oncomine.org by following the link. TCGA breast data was obtained through the UCSC cancer browser (genome-cancer.ucsc.edu).

### GSEA analysis

Correlation was performed comparing AR vs. all other genes in the TN breast cancer samples from TCGA and the Servant data set. The Spearman’s correlation coefficient rho was generated by correlating AR vs. each other gene. This was put into GSEA using the pre-ranked algorithm and run with the C2:curated gene sets.

### Statistical analysis

Data are presented as the mean ± SD or SEM of at least three experiments as indicated. Statistical significance was determined using Student’s *t*-test, and *p*<0.05 were considered statistically different. Pearson’s correlation method was used to assess correlation between factors. As with previously reported studies, analysis of variance was utilized to evaluate factors of treatment (radiation sensitivity, AR inhibition, and radiation) individually and jointly.^26^ The *F*-test was used to test for significance of factors. When statistically significant interactions were found, the Tukey Honest Significant Difference method was used to compare differences in mean tumor volume for all pairs of treatment groups. All tests were conducted at α = 0.05. All other statistical analyses were performed as described in the text. Kaplan–Meier curves were generated, and univariate and multivariate analysis was performed using Cox regression. Univariable and Multivariable analyses were run using MedCalc 15 software. AR was analyzed as a continuous variable. Analysis using AR as an ordinal value was also performed but not shown.

### Data availability

Data supporting the claims in this paper is present within the article as well as the Supplemental files. The Servant data set was downloaded from EMBL-EBI Array Expression repository (https://www.ebi.ac.uk/arrayexpress/). Curtis and van de Vijver data sets were downloaded from Oncomine (https://www.oncomine.org/resource/login.html). TCGA breast data set was obtained through the TCGA Data Portal (now reposited at Genomic Data Commons of the NCI- https://gdc.cancer.gov/).

## Electronic supplementary material

Supplementary Figure 4 Full western blot scan

Figure 6 Full western blot scan

Supplementary Figure 2 Full western blot scan

Supp Figure and Table Legends

Supplemental Figure 1

Supplemental Figure 2

Supplemental Figure 3

Supplemental Figure 4

Supp Figure 5

Supplemental Table 1

Supplemental Table 2

Supplemental Table 3
