# Supplementary figures and images for: Androgen receptor as a mediator and biomarker of radioresistance in triple-negative breast cancer
Source: NPJ Breast Cancer. 2017 Aug 18;3:29. doi: 10.1038/s41523-017-0038-2 (PMC5562815; doi:10.1038/s41523-017-0038-2)

for scan image

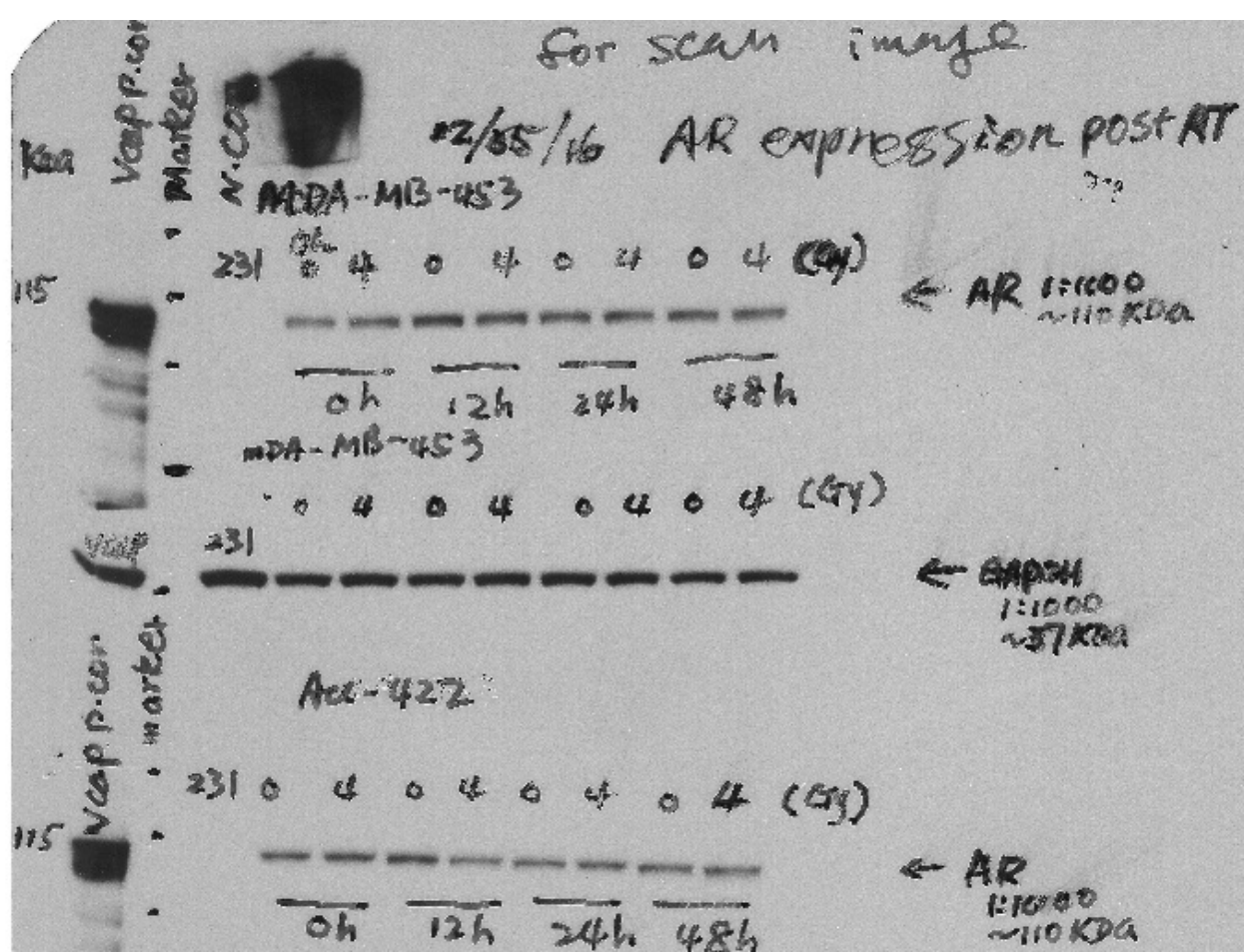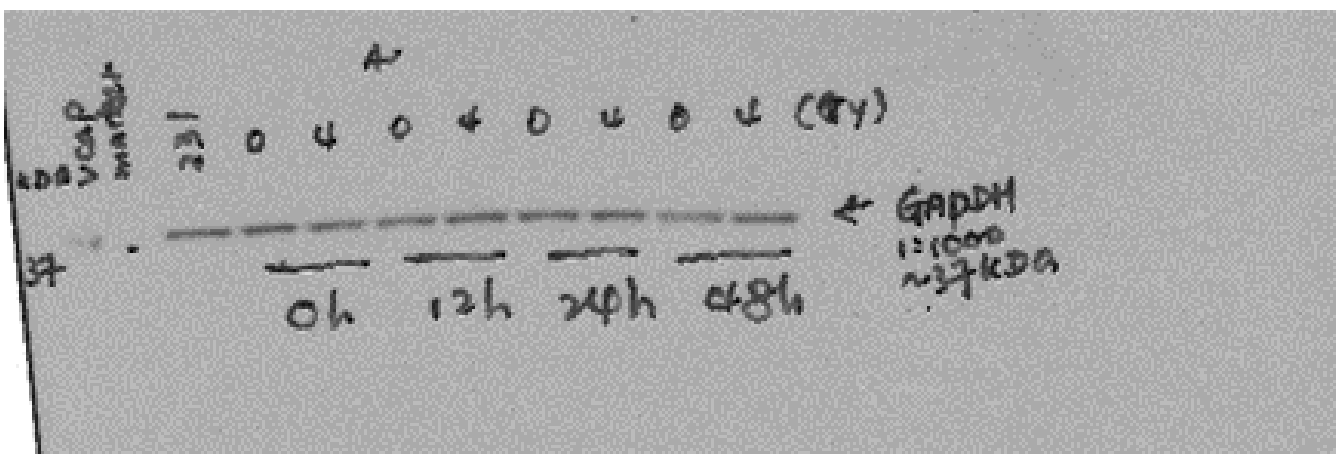

Supplement: Supplementary file 1 — Supplementary Figure 4 Full western blot scan [file 41523_2017_38_MOESM1_ESM.pdf]

$\alpha$  AR \*

|||||

11/4/16

MDA-231

MDA-453

SUM-185

MCF7

BT 549

HCC 3B

HCC 70

HCC 460

HCC 1561

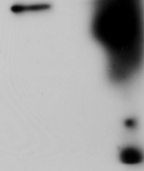

GAPDH

|||||

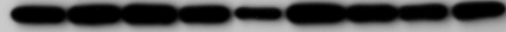

AR blot reprobed

11/7/16

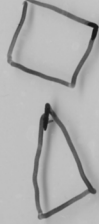

Supplement: Supplementary file 3 — Supplementary Figure 2 Full western blot scan [file 41523_2017_38_MOESM3_ESM.pdf]

Supplemental Figure 1

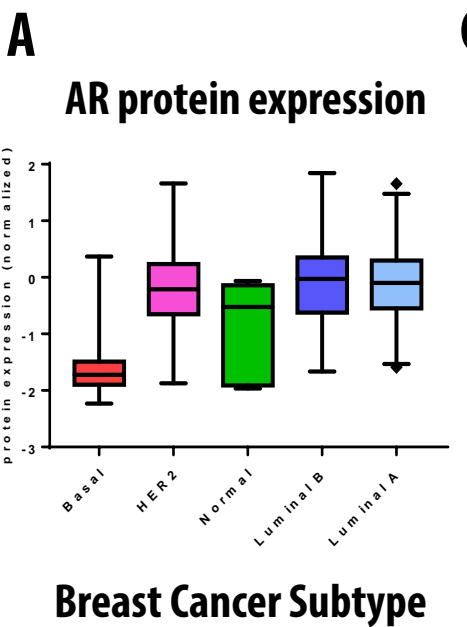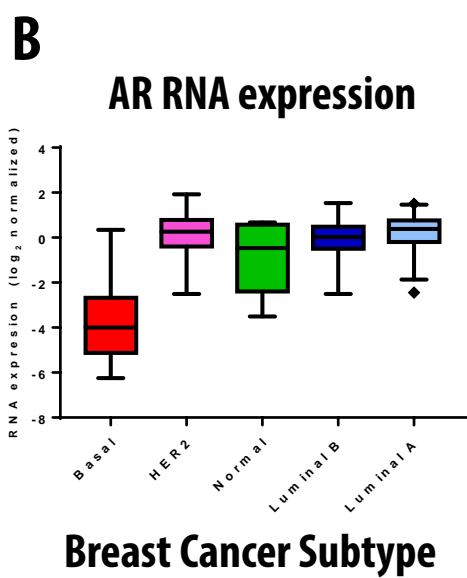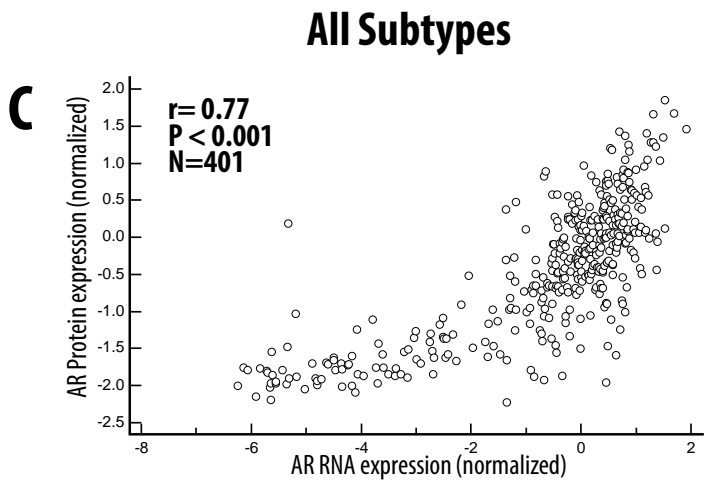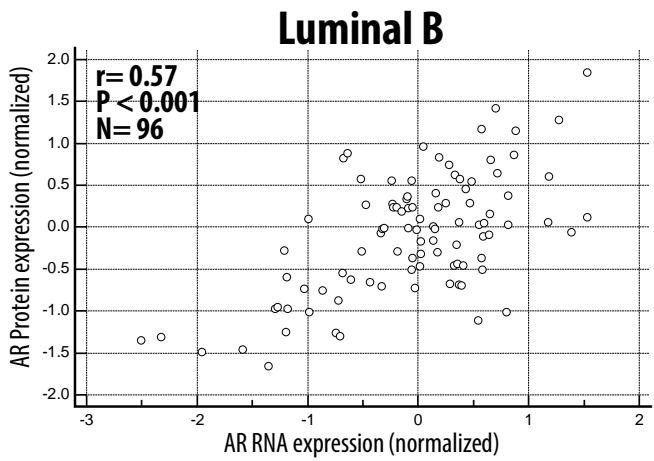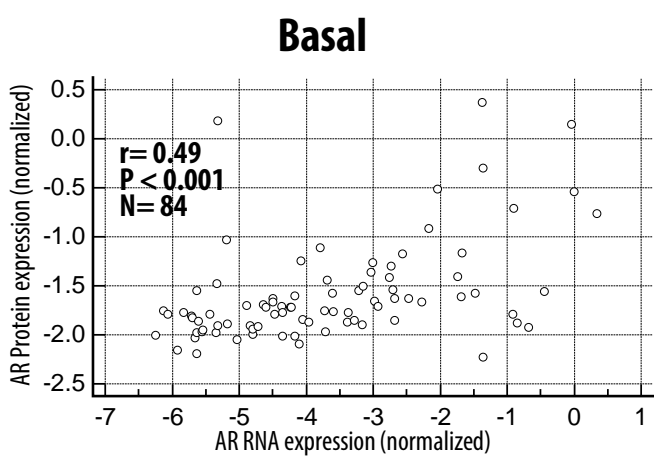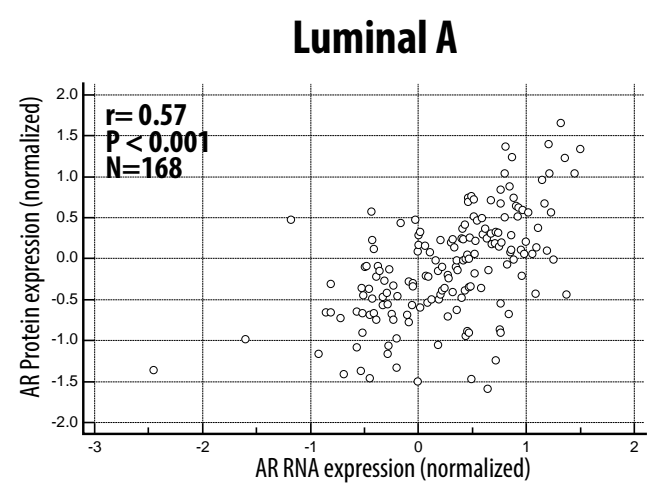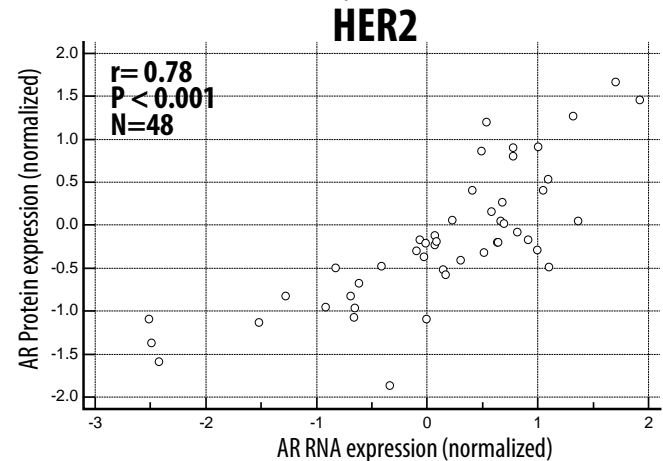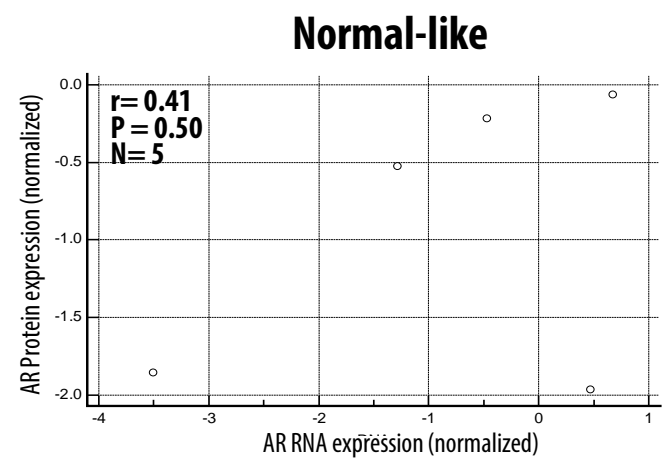

Supplement: Supplementary file 5 — Supplemental Figure 1 [file 41523_2017_38_MOESM5_ESM.pdf]

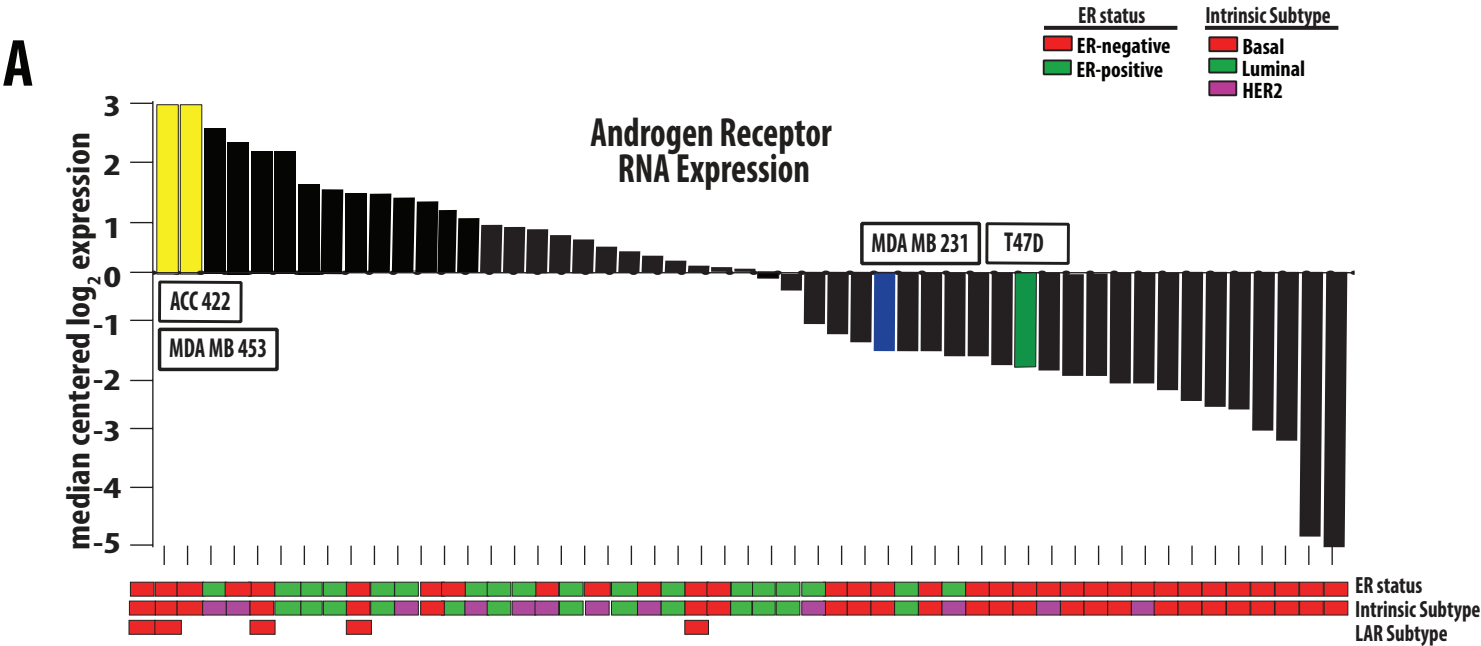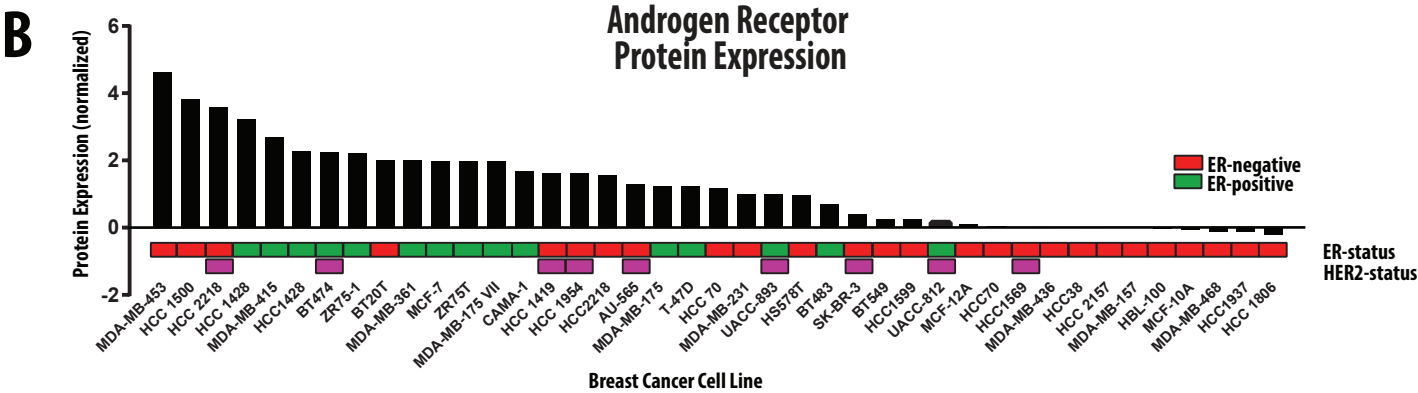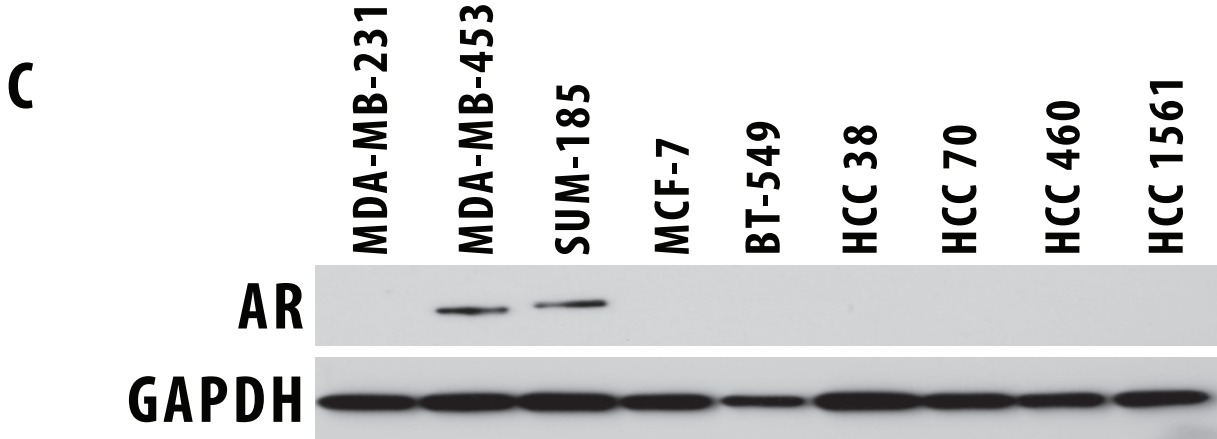

Supplement: Supplementary file 6 — Supplemental Figure 2 [file 41523_2017_38_MOESM6_ESM.pdf]

Supplementary Figure 4

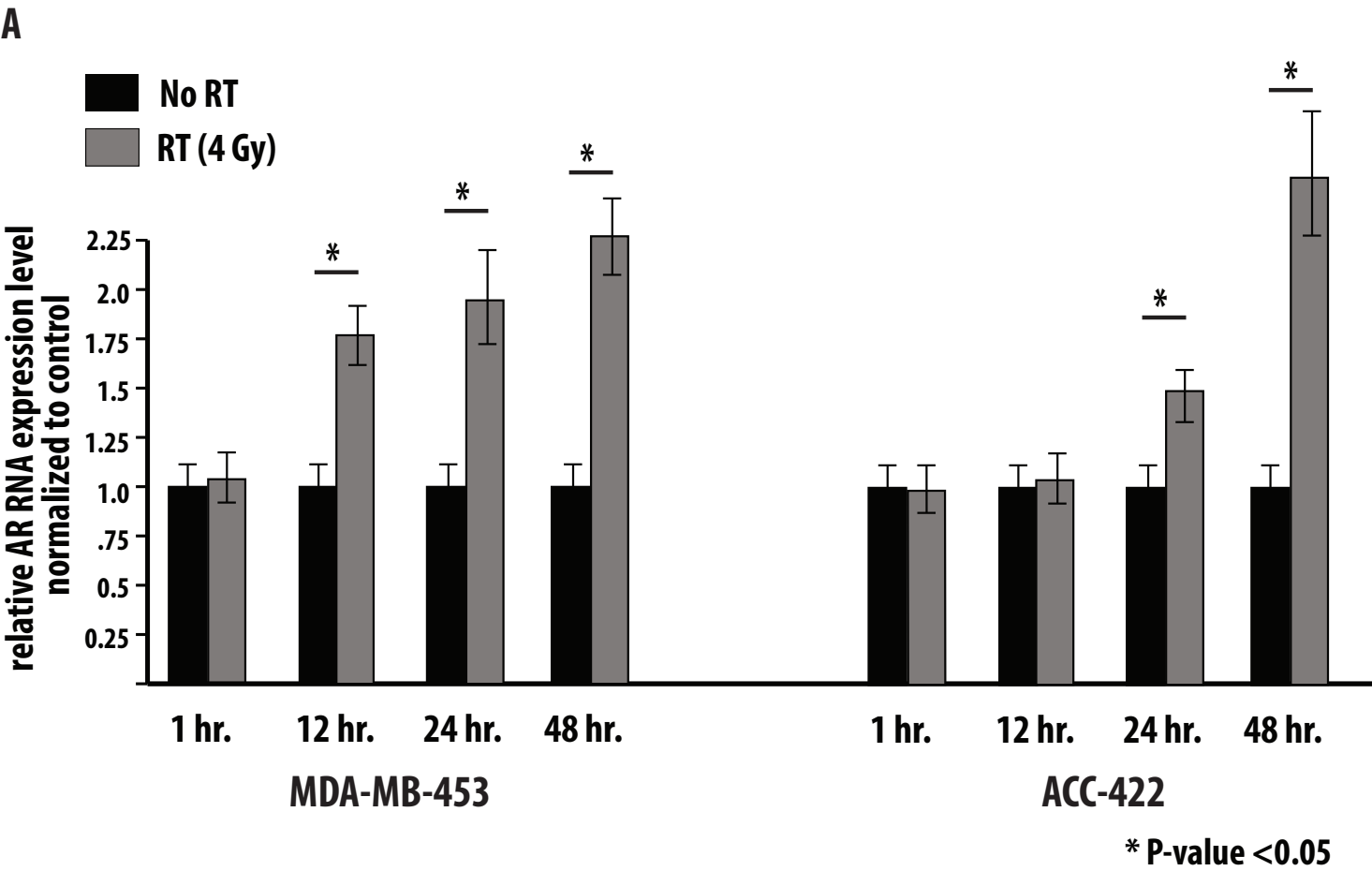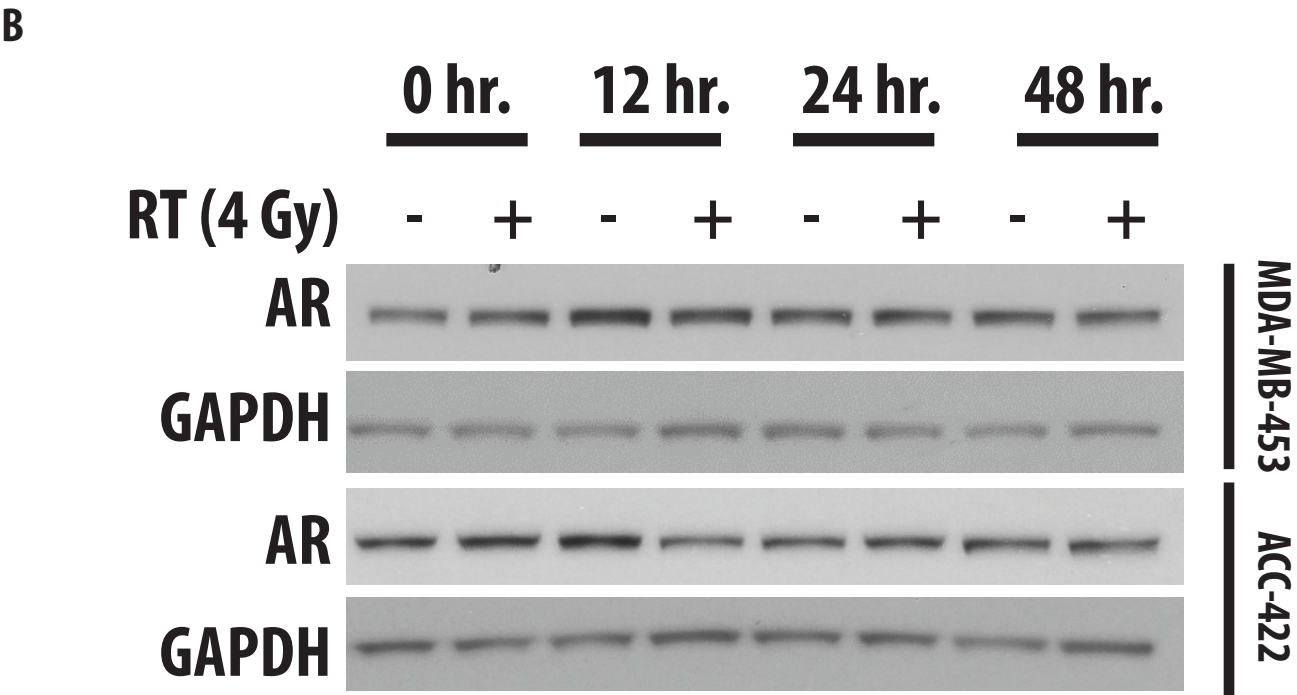

Supplement: Supplementary file 8 — Supplemental Figure 4 [file 41523_2017_38_MOESM8_ESM.pdf]

Supplementary Figure 5

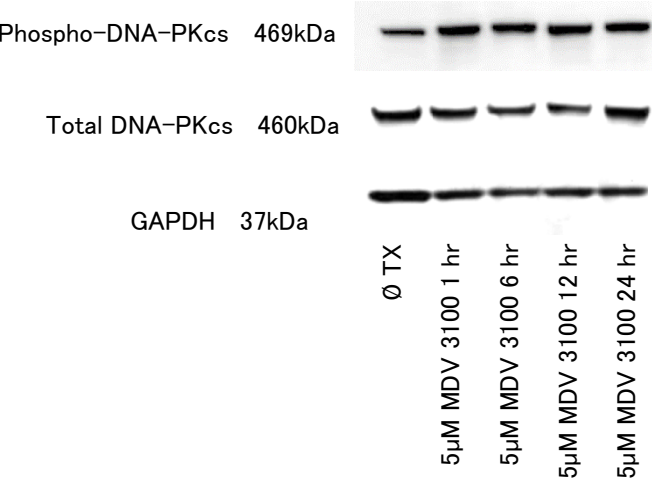

Supplement: Supplementary file 9 — Supp Figure 5 [file 41523_2017_38_MOESM9_ESM.pdf]
