# Supplementary material for: Androgen receptor as a mediator and biomarker of radioresistance in triple-negative breast cancer
Source: NPJ Breast Cancer. 2017 Aug 18;3:29. doi: 10.1038/s41523-017-0038-2 (PMC5562815; doi:10.1038/s41523-017-0038-2)
Supplement: Supplementary file 2 — Figure 6 Full western blot scan [file 41523_2017_38_MOESM2_ESM.pdf]

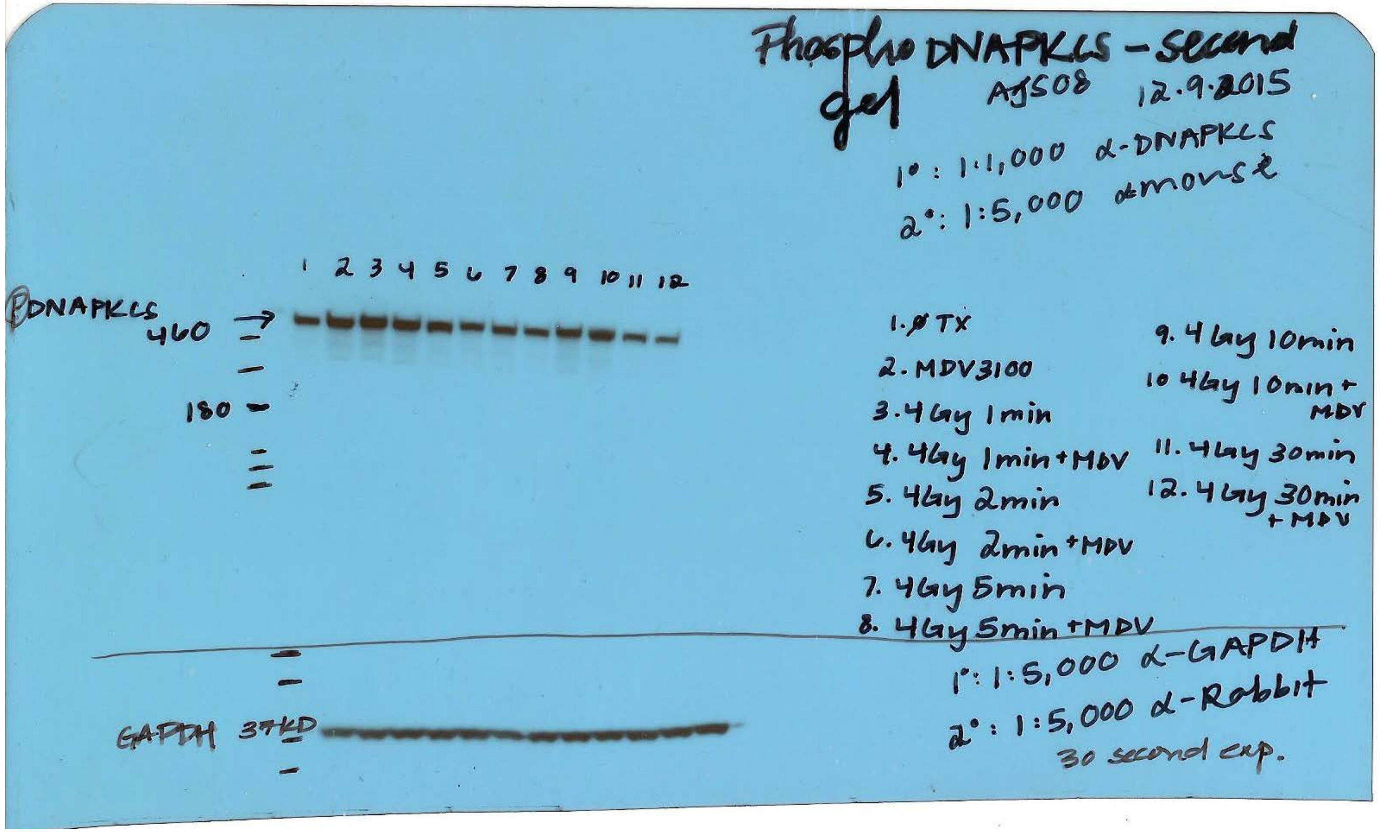
Phospho DNAPKcs - second gel
AJS08 12.9.2015
1°: 1:1,000 α-DNAPKcs
2°: 1:5,000 αmouse
1 2 3 4 5 6 7 8 9 10 11 12
ⓟDNAPKcs
460
180
1. ØTX
2. MDV3100
3. 4Gy 1min
4. 4Gy 1min+MDV
5. 4Gy 2min
6. 4Gy 2min+MDV
7. 4Gy 5min
8. 4Gy 5min+MDV
9. 4Gy 10min
10 4Gy 10min+MDV
11. 4Gy 30min
12. 4Gy 30min +MDV
1°: 1:5,000 α-GAPDH
2°: 1:5,000 α-Rabbit
30 second exp.
GAPDH 37KD

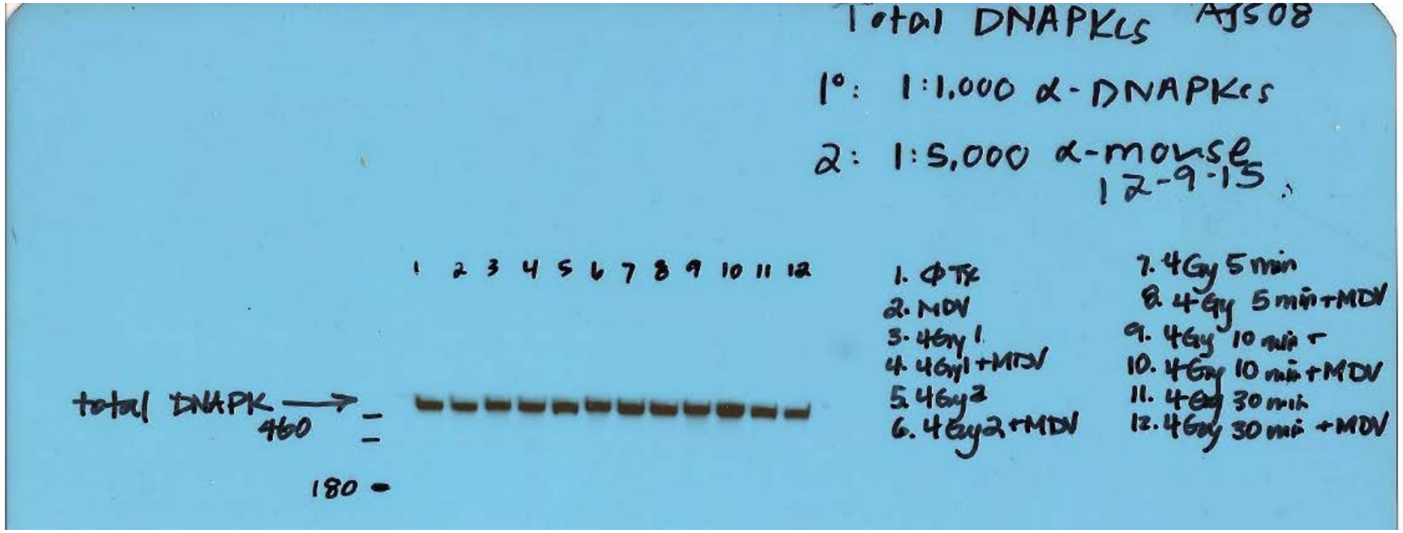
Total DNAPKcs AJS08
1°: 1:1,000 α-DNAPKcs
2: 1:5,000 α-mouse
12-9-15
1 2 3 4 5 6 7 8 9 10 11 12
1. ØTx
2. MDV
3. 4Gy 1
4. 4Gy 1 +MDV
5. 4Gy 2
6. 4Gy 2 +MDV
7. 4Gy 5 min
8. 4Gy 5 min +MDV
9. 4Gy 10 min +
10. 4Gy 10 min +MDV
11. 4Gy 30 min
12. 4Gy 30 min +MDV
total DNAPK
460
180
