# Supplementary material for: Androgen receptor as a mediator and biomarker of radioresistance in triple-negative breast cancer
Source: NPJ Breast Cancer. 2017 Aug 18;3:29. doi: 10.1038/s41523-017-0038-2 (PMC5562815; doi:10.1038/s41523-017-0038-2)
Supplement: Supplementary file 4 — Supp Figure and Table Legends [file 41523_2017_38_MOESM4_ESM.pdf]

Supplementary Figure 1- AR expression in ~2,000 BC tumors from patients demonstrates heterogeneity in protein (A) and RNA (B) expression with no significant correlation to ER status or breast cancer subtype. There is a strong correlation between RNA and protein AR expression in BC tumor samples across all intrinsic subtypes of breast cancer (C). Correlation coefficients (R), p-values, and total number of samples per group (N) are noted.

Supplementary Figure 2- AR RNA and protein expression is also heterogeneous in breast cancer cell lines. The triple-negative breast cancer cell lines identified as being in the "luminal androgen receptor" subtype of TNBC have higher levels of AR-expression, but are not the only breast cancer cell lines that demonstrated AR RNA and protein expression (A, B). RNA expression was measured with Affymetrix microarrays (A) and protein expression was measured by reverse phase protein lysate array (B) or western blotting (C).

Supplementary Figure 3- On target effects of MDV3100 in vivo in tumors treated with radiation +/- MDV3100. AR and AR-target genes (AQP3, SEC14L2, and PRKDC) are listed. Error bars represent S.D.

Supplementary Figure 4- AR RNA expression is upregulated after radiation in MDA-MB-453 and ACC-422 cells (A) with RNA measured with or without radiation at 1, 12, 24, and 48 hrs. AR protein levels were measured at similar time points by western blotting (B) and remain unchanged in the same cell lines.

Supplementary Figure 5- total and phospho-DNAPKcs expression is unchanged at various time points with MDV3100 treatment. Total and phospho-DNAPKcs levels were measured after pretreatment with MDV3100. GAPDH was used as a protein loading control.

Supplementary Table 1- Intrinsic radiation sensitivity values in 21 breast cancer cell lines as measured by clonogenic survival assay. Values depicted as surviving fraction after 2 Gy (SF 2-Gy) with the associated breast cancer cell line instrinsic subtype as described by Neve et al.

Supplementary Table 2- A list of all drugs, targets, and PubCHEM IDs for the drugs used in the radiosensitizer screen.

Supplementary Table 3- Clinicopathologic characteristics of the patients from the Servant et al. and vande Vijver et al. datasets.
