# Supplementary material for: Androgen receptor as a mediator and biomarker of radioresistance in triple-negative breast cancer
Source: NPJ Breast Cancer. 2017 Aug 18;3:29. doi: 10.1038/s41523-017-0038-2 (PMC5562815; doi:10.1038/s41523-017-0038-2)
Supplement: Supplementary file 7 — Supplemental Figure 3 [file 41523_2017_38_MOESM7_ESM.pdf]

Supplementary Figure 3

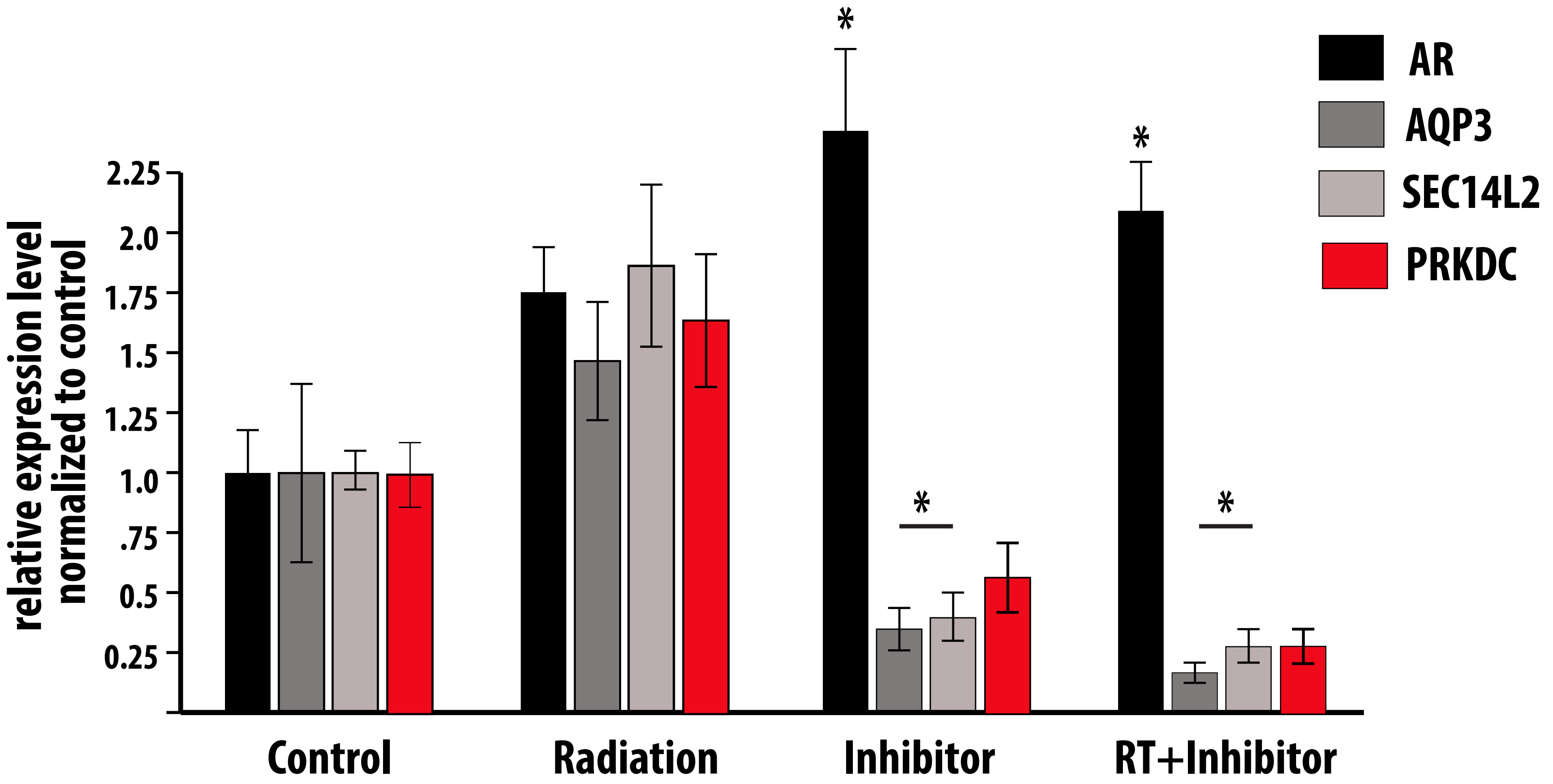

MDA-MB-453 cells
MDV3100 10 mg/kg/day
RT: 5 fractions, 2 Gy/day
4 tumors pooled, 16 tumors per group

* P-value <0.05
