# Supplementary material for: Androgen receptor as a mediator and biomarker of radioresistance in triple-negative breast cancer
Source: NPJ Breast Cancer. 2017 Aug 18;3:29. doi: 10.1038/s41523-017-0038-2 (PMC5562815; doi:10.1038/s41523-017-0038-2)
Supplement: Supplementary file 10 — Supplemental Table 1 [file 41523_2017_38_MOESM10_ESM.pdf]

Supplementary Table 1

Cell line	SF-2Gy	Intrinsic subtype*
BT-549	0.772	BaB
MDA-MB-453	0.684	Lu
BT-20	0.606	BaA
HCC-38	0.570	BaB
MDA-MB-231	0.546	BaB
AU565	0.534	Lu
HCC-1954	0.497	HER2
T47D	0.475	Lu
HCC-1806	0.447	NA
CAMA-1	0.434	Lu
ACC-440	0.430	BaA
ACC-459	0.393	BaB
ACC-231	0.390	Lu
MCF-7	0.379	Lu
ACC-302	0.355	BaB
MDA-MB-361	0.334	HER2
HCC-70	0.329	BaA
BT474	0.308	Lu
ZR75.30	0.301	HER2
HCC-1937	0.180	BaA
ACC-422	0.174	Lu

* Neve et al., Cancer Cell. 2006 Dec;10(6):515-27.
