# Supplementary material for: Androgen receptor as a mediator and biomarker of radioresistance in triple-negative breast cancer
Source: NPJ Breast Cancer. 2017 Aug 18;3:29. doi: 10.1038/s41523-017-0038-2 (PMC5562815; doi:10.1038/s41523-017-0038-2)
Supplement: Supplementary file 11 — Supplemental Table 2 [file 41523_2017_38_MOESM11_ESM.pdf]

Supplementary Table 2- All drugs used in screen

Name	Synonyms	Targets	Target pathway	PubCHEM
(5Z)-7-Oxozeaenol		TAK1 (MAP3K7)	other	
17-AAG	tanespimycin	HSP90	other	6505803
5-Fluorouracil	5-FU	DNA antimetabolite	DNA replication	
681640	681640	WEE1, CHEK1	cell cycle	10384072
A-443654	KIN001-139	AKT1, AKT2, AKT3	PI3K signaling	10172943
A-770041	KIN001-111	SRC family	other	9549184
AC220	Quizartinib	FLT3	RTK signaling	24889392
AG-014699	PF-01367338	PARP1, PARP2	Genome integrity	9931953
AICAR	N1-(b-D-Ribofuranosyl)-5-aminoimidazole-4-carboxamide	AMPK agonist	metabolism	65110
AKT inhibitor VIII		AKT1, AKT2, AKT3	PI3K signaling	16218954
AMG-706	AMG-706	VEGFR, RET, c-KIT, PDGFR	RTK signaling	11667893
AP-24534	KIN001-192	ABL	ABL signaling	24826799
AR-42	HDAC-42	HDAC	chromain histone acetylation	6918848
AS601245	AS601245	JNK	JNK and p38 signaling	11422035
AS605240	KIN001-173	PI3K gamma	PI3K signaling	
AT-7519		CDK9	cell cycle	
ATRA	Tretinoin	Retinoic acid and retinoid X receptor agonist	other	444795
AUY922	VER-52296,NVP-AUY922	HSP90	other	10096043
AZ628		BRAF	ERK MAPK signaling	11676786
AZD6482	KIN001-193	PI3K beta (P3C2B)	PI3K signaling	44137675
AZD6482	WO2009093972	PI3K beta (P3C2B)	PI3K signaling	
AZD7762	AZD 7762	CHEK1, CHEK2	Genome integrity	
AZD8055	AZD8055	mTORC1/2	TOR signaling	
Afatinib	BIBW2992, Tovok	ERBB2, EGFR	EGFR signaling	10184653
Afatinib		ERBB2, EGFR	EGFR signaling	
Axitinib	AG-013736	PDGFR, KIT, VEGFR	RTK signaling	6450551
BAY 61-3606		SYK	other	16760671
BEZ235	NVP-BEZ235	PI3K (class 1), mTORC1/2	PI3K signaling	
BHG712	NVP-BHG712	EPHB4	RTK signaling	16747388
BI-2536	KIN001-124	PLK1, PLK2, PLK3	mitosis	11364421
BIRB 0796		Androgen receptor (ANDR)	JNK and p38 signaling	156422
BIX02189		MEK5	other	46931012
BMS-509744	KIN001-127	ITK	other	20635522
BMS-536924	KIN001-126	IGF1R	IGFR signaling	10390396
BMS-536924	BMS-536924	IGF1R	IGFR signaling	
BMS-708163	Avagacestat	gamma-secretase	other	
BMS-708163	Avagacestat	gamma-secretase	other	46883536
BMS-754807		IGF1R	IGFR signaling	24785538
BMS345541	BMS345541	IKBKB	other	
BX-795	BX 795	TBK1, PDPK1, IKK, AURKB, AURKC	other	10077147
BX-912	KIN001-175	PDK1 (PDPK1)	PI3K signaling	

Belinostat	PXD101	HDAC	chromain histone acetylation	6918638
Bexarotene	LG-100069,LGD-1069	Retinioic acid X family	other	82146
Bicalutamide	ICI-176334	Androgen receptor (ANDR)	other	2375
Bicalutamide	ICI-176334	Androgen receptor (ANDR)	other	
Bleomycin		DNA damage	DNA replication	5460769
Bleomycin (50 uM)		DNA damage	DNA replication	
Bortezomib	LDP-341,PS-341	Proteasome	other	387447
Bosutinib	SKI-606	SRC, ABL, TEC	ABL signaling	5328940
Bryostatin 1	Bryostatin 1	PRKC	other	5280757
CAL-101		PI3K delta	PI3K signaling	
CAY10603		HDAC6	chromain histone acetylation	24951314
CCT007093		PPM1D	other	
CCT018159		HSP90	other	
CEP-701	CEP-701	FLT3, JAK2, NTRK1, RET	RTK signaling	126565
CGP-082996	CINK4,KIN001-021	CDK4	cell cycle	24825971
CGP-60474	KIN001-019	CDK1,CDK2,CDK5,CDK7,CDK9	cell cycle	644215
CH5424802		ALK	RTK signaling	49806720
CHIR-99021	CT 99021	GSK3B	WNT signaling	9956119
CHIR-99021	CT 99021	GSK3B	WNT signaling	9956119
CI-1040	PD-18435,PD-184352	MEK1, MEK2	ERK MAPK signaling	6918454
CMK	KIN001-128	RSK	ERK MAPK signaling	NO MATCH
CP466722	[2-(6,7-dimethoxyquinazolin-4-yl)-5-(pyridin-2-yl)-2H-1,2,4-triazol-3-amine]	ATM	Genome integrity	44551660
CP724714		ERBB2	EGFR signaling	9874913
CUDC-101		HDAC, EGFR	chromain histone acetylation	24756910
CX-5461		RNA Pol I	other	25257557
Camptothecin	7-Ethyl-10-Hydroxy-Camptothecin,SN-38	TOP1	DNA replication	104842
Cetuximab	Erbitux	EGFR	EGFR signaling	
Cisplatin	cis-Diammineplatinum(II) dichloride	DNA crosslinker	DNA replication	84691
Crizotinib	Xalkori,PF2341066	MET, ALK	RTK signaling	11496366
Cyclopamine	11-deoxojervine	SMO	other	442972
Cytarabine	Ara-Cytidine,Arabinosyl Cytosine,U-19920	DNA synthesis	DNA replication	6253
DMOG	Dimethyloxalylglcine	Prolyl-4-Hydroxylase	other	560326
Dabrafenib	GSK2118436	BRAF	ERK MAPK signaling	
Dasatinib	KIN001-005	ABL, SRC, KIT, PDGFR	ABL signaling	3062316
Docetaxel	RP-56976	Microtubules	cytoskeleton	148124
Doxorubicin	Doxil,Rubex	DNA intercalating	DNA replication	31703
EHT 1864		Rac GTPases	cytoskeleton	
EKB-569	Pelitinib	EGFR	EGFR signaling	6445562
EX-527		SIRT1	other	5113032
Elesclomol		HSP70	other	
Embelin		XIAP	apoptosis regulation	3218

Epothilone B	GNF-PF-193	Microtubules	cytoskeleton	448013
Erlotinib		EGFR	EGFR signaling	176870
Etoposide		TOP2	DNA replication	36462
FH535	FH535	unknown	other	3463933
FK866	APO866	NAMPT inhibitor	metabolism	
FMK	KIN001-242	RSK	ERK MAPK signaling	
FR-180204		ERK	ERK MAPK signaling	11493598
FTI-277		Farnesyl-transferase (FNTA)	other	3005532
Foretinib	GSK1363089, XL-880	MET	RTK signaling	42642645
GDC0449	GDC0449	SMO	other	24776445
GDC0941		PI3K (class 1)	PI3K signaling	17755052
GDC0941		PI3K	PI3K signaling	
GNF-2	KIN001-013 (GNF-2 / 3-(6-(4-(trifluoromethoxy)phenylamino)pyrimidin-4-yl)benzamide)	ABL (T315I)	ABL signaling	5311510
GSK-650394		SGK3	other	25022668
GSK1070916		AURKB	mitosis	46885626
GSK1904529A	GSK1904529A	IGF1R	IGFR signaling	25124816
GSK2126458	EX-8678	PI3K, mTOR	PI3K signaling	52914946
GSK269962A	KIN001-155	ROCK1,ROCK2	cytoskeleton	16095342
GSK269962A	KIN001-155	ROCK1,ROCK2	cytoskeleton	1609532
GSK429286A		ROCK2	cytoskeleton	
GSK690693		AKT	PI3K signaling	16725726
GW 441756		NTRK1	RTK signaling	
GW-2580	GW-2580	cFMS	RTK signaling	
GW843682X	KIN001-134	PLK1	mitosis	15983966
Gefitinib	ZD-1839	EGFR	EGFR signaling	123631
Gemcitabine	Gemzar, LY-188011	DNA replication	DNA replication	60750
Genentech Cpd 10		AURKA, AURKB	mitosis	
HG-5-113-01		LOK, LTK, TRCB, ABL(T315I)	ABL signaling	
HG-5-88-01		EGFR, ADCK4	EGFR signaling	
HG-6-64-1	KIN001-206	BRAFV600E, TAK, MAP4K5	ERK MAPK signaling	
I-BET-762	GSK525762A	BRD2, BRD3, BRD4	chromatin other	46943432
IOX2		EGLN1	other	
IPA-3	IPA-3	PAK	cytoskeleton	521106
Imatinib	Gleevec,STI-571	ABL, KIT, PDGFR	ABL signaling	5291
Ispinesib Mesylate	SB-715992	KIF11	mitosis	6851740
JNJ-26854165		MDM2	p53 pathway	11609586
JNK Inhibitor VIII	JNK Inhibitor VIII	JNK	JNK and p38 signaling	11624601
JNK-9L	KIN001-204	JNK, CDK9	JNK and p38 signaling	NO MATCH
JQ1	JQ1	BRD2, BRD3, BRD4	chromatin other	
JQ1		BRD2, BRD3, BRD4	chromatin other	46907787
JQ12	JQ12	HDAC	chromain histone acetylation	
JW-7-24-1		LCK	other	
JW-7-52-1		mTOR	TOR signaling	49836027
KIN001-055	WHI-P97, AC1L1GQE	JAK3, MNK1	other	3796
KIN001-102		AKT1	PI3K signaling	
KIN001-135	KIN001-135	IKKE	other	11626927

KIN001-236		TIE2	other	
KIN001-244		PDK1 (PDPK1)	PI3K signaling	56965967
KIN001-260	Bayer IKKb inhibitor	IKK	other	
KIN001-266		TPL2,COT(M3K8)	other	44143370
KIN001-270		CDK9	cell cycle	
KU-55933		ATM	Genome integrity	5278396
LAQ824	Dacinostat, NVP-LAQ824	HDAC	chromain histone acetylation	6445533
LFM-A13	DDE-28	BTK	other	9549280
LY317615	Enzastaurin	PKCbeta	other	176167
Lapatinib	Tykerb, Tyverb	EGFR, ERBB2	EGFR signaling	208908
Lenalidomide		TNF alpha	other	216326
Linifanib	ABT-869	RTK	RTK signaling	11485656
Lisitinib	OSI-906	IGF1R	IGFR signaling	11640390
MG-132	zLLL	Proteasome	other	462382
MK-2206		AKT1, AKT2	PI3K signaling	
MLN4924		NEDD8-activating enzyme (NAE)	other	
MP470		PDGFR	RTK signaling	11282283
MPS-1-IN-1		MPS1	mitosis	25195352
MS-275	MS275	HDAC	chromain histone acetylation	4261
Masitinib	AB1010	KIT	RTK signaling	10074640
Methotrexate		Dihydrofolate reductase	DNA replication	126941
Midostaurin	PKC 412	KIT	RTK signaling	24202429
Mitomycin C	Mytozytrex,NSC-26980	DNA crosslinker	DNA replication	5746
NG-25		TAK	other	
NPK76-II-72-1		PLK3	mitosis	46843648
NSC-207895	XI-006	MDMX	p53 pathway	3246561
NSC-87877		SHP-1 (PTPN6), SHP-2	other	5459322
NU-7441	KU-57788	DNAPK	Genome integrity	11327430
Navitoclax	ABT-263	BCL-2, BCL-XL, BCL-W	apoptosis regulation	24978538
Nilotinib		ABL	ABL signaling	644241
Nutlin-3a (-)	Nutlin-3a (-)	MDM2	p53 pathway	11433190
OSI-027	A-1065	mTORC1/2	TOR signaling	44224160
OSI-930		KIT, VEGFR, PDGFR	RTK signaling	9868037
OSU-03012	AR-12	PDK1 (PDPK1)	PI3K signaling	10027278
Obatoclax Mesylate		BCL-2, BCL-XL, MCL-1	apoptosis regulation	16727411
Olaparib	KU0059436, AZD-2281	PARP1, PARP2	Genome integrity	23725625
Olaparib	KU0059436, AZD2281	PARP1, PARP2	Genome integrity	
PAC-1	PAC-1	CASP3 agonist	apoptosis regulation	6851947
PD-0325901	PD-0325901	MEK1, MEK2	ERK MAPK signaling	9826528
PD-0332991	Palbociclib Isethionate	CDK4, CDK6	cell cycle	5330286
PD-173074	PD-173074	FGFR1, FGFR3	RTK signaling	1401
PF-4708671		p70 S6KA	TOR signaling	
PF-562271	KIN001-205	FAK	cytoskeleton	11713159
PFI-1		BRD2, BRD3, BRD4	chromatin other	
PHA-665752		MET	RTK signaling	10461815
PHA-793887		CDK-pan	cell cycle	46191454

PI-103		PI3K alpha,DNAPK	PI3K signaling	9884685
PIK-93		PI4K,PI3K	PI3K signaling	6852167
PLX4720		BRAF	ERK MAPK signaling	24180719
PLX4720		BRAF	ERK MAPK signaling	
Paclitaxel	BMS-181339-01	Beta subunit of Tubulin	cytoskeleton	36314
Parthenolide		NFKB1	other	5420805
Pazopanib	GW786034	VEGFR, PDGFRA, PDGFRB, KIT	RTK signaling	10113978
Phenformin	Phenformin		metabolism	
Piperlongumine		Increases ROS levels	other	637858
Pyrimethamine		Dihydrofolate reductase	DNA replication	4993
QL-VIII-58		mTOR, ATR	TOR signaling	
QL-X-138		MNK2, DNAPK, MTOR, BTK,	other	
QL-XI-92		DDR1	RTK signaling	
QL-XII-47		BTK, BMX	other	
QL-XII-61		BMX, BTK	other	
QS11	QS11	ARFGAP	other	No Match
RDEA119	RDEA119	MEK1, MEK2	ERK MAPK signaling	44182295
RDEA119		MEK1, MEK2	ERK MAPK signaling	
RO-3306		CDK1	cell cycle	44450571
Rapamycin	AY-22989,Sirolimus,WY-090217	mTOR	TOR signaling	5284616
Roscovitine	Seliciclib	CDK family	cell cycle	160355
Ruxolitinib	INCB-18424	JAK1, JAK2, TYK2	other	25127112
S-Trityl-L-cysteine	NSC 83265	KIF11	mitosis	76044
SB 216763	SB 216763	GSK3A, GSK3B	WNT signaling	
SB 505124	2-(5-Benzo[1,3]dioxol-5-yl-2-tert-butyl-3H-imidazol-4-yl)-6-methylpyridine hydrochloride hydrate	TGFbetaR-I (ALK5)	other	24724611
SB52334		ALK5	RTK signaling	9967941
SB590885		BRAF	ERK MAPK signaling	11316960
SGC0946		DOT1L (Q8TEK3)	chromatin histone methylation	
SL 0101-1		RSK, AURKB, PIM3	ERK MAPK signaling	
SN-38	7-ethyl-10-hydroxy-camptothecin	TOP1	DNA replication	
SNX-2112		HSP90	other	24772860
STF-62247		Autophagy	other	704473
Salubrinal	3-Phenyl-N-[2,2,2-trichloro-1-[[(8-quinolinylamino)thioxomethyl]amino]ethyl]-2-propenamide	GADD34-PP1C phosphatase	other	5717801
Saracatinib	AZD-0530,KIN001-045	SRC, ABL1	ABL signaling	10302451
Selumetinib		MEK1, MEK2	ERK MAPK signaling	10127622
Selumetinib		MEK1, MEK2	ERK MAPK signaling	
Shikonin	Shikonin	unknown	other	5208
Sorafenib	BAY-439006, Nexavar	PDGFRA, PDGFRB, KDR, KIT, FLT3	RTK signaling	216239
Sunitinib	Sutent	PDGFRA, PDGFRB, KDR, KIT, FLT3	RTK signaling	5329102

T0901317		LXR	other	447912
TAE684	NVP-TAE684	ALK	RTK signaling	16038120
TAK-715	KIN001-201	p38a	JNK and p38 signaling	
TG101348		JAK2	other	16722836
TGX221		PI3K beta	PI3K signaling	9907093
THZ-2-102-1		CDK7	cell cycle	
THZ-2-49		CDK9	cell cycle	
TL-1-85		TAK	other	
TL-2-105		CRAF	ERK MAPK signaling	
TPCA-1		IKK	other	9903786
TW 37		BCL-2, BCL-XL	apoptosis regulation	11455910
Talazoparib	BMN-673	PARP1, PARP2	Genome integrity	
Tamoxifen		ER	other	
Temozolomide	Temodar	DNA akylating agent	DNA replication	
Temsirolimus	CCI-779	mTOR	TOR signaling	6918289
Thapsigargin		ATPase, Ca++ transporting, cardiac muscle, slow twitch 2	other	446378
Tipifarnib		Farnesyl-transferase (FNTA)	other	159324
Tivozanib	AV-951	VEGFR	RTK signaling	9911830
Trametinib	GSK1120212	MEK1, MEK2	ERK MAPK signaling	
Tubastatin A		HDAC6	chromain histone acetylation	53394750
UNC0638		G9a(EHMT2), GLP(EHMT1)	chromatin histone methylation	46224516
UNC0638		G9a(EHMT2), GLP(EHMT1)	chromatin histone methylation	
UNC1215		L3MBTL3	other	
VNLG/124	4-(Butanoyloxymethyl)phenyl-(2E,4E,6E,8E)-3,7-dimethyl-9-(2,6,6-trimethylcyclohex-1-enyl)nona-2,4,6,8-tetraenoate	HDAC,RAR	chromain histone acetylation	24894414
VX-11e		ERK	ERK MAPK signaling	11634725
VX-680	MK-045,MK-0457,VX-68	AURKA, AURKB, AURKC, FLT3, ABL1, JAK2	mitosis	5494449
VX-702		p38	JNK and p38 signaling	10341154
Veliparib	ABT-888	PARP1, PARP2	Genome integrity	11960529
Vinblastine	Vinblastine sulphate	Microtubules	cytoskeleton	6710780
Vinorelbine		Microtubules	cytoskeleton	60780
Vorinostat	SAHA	HDAC inhibitor Class I, IIa, IIb, IV	chromain histone acetylation	5311
WH-4-023	KIN001-112	SRC family, ABL	ABL signaling	11844351
WZ-1-84	KIN001-123	BMX	other	49821040
WZ3105		CLK2, CNSK1E, FLT3, ULK1	other	
XAV939	NVP-XAV939	TNKS1, TNKS2	WNT signaling	
XL-184	Cabozantinib	VEGFR, MET, RET, KIT, FLT1, FLT3, FLT4, TIE2,AXL	RTK signaling	25102847
XMD11-85h		BRSK2, FLT4, MARK4, PRKCD, RET, SPRK1	other	
XMD13-2		RIPK	other	

XMD14-99		EPHB3, CAMK1	RTK signaling	
XMD15-27		CAMK2B, CLK2, DYRK1A, MAST1, STK39	other	
XMD8-85	XMD8-85	ERK5 (MK07)	other	46844147
XMD8-92		ERK5	other	
Y-39983		ROCK	cytoskeleton	9810884
YK 4-279		RNA helicase A	other	
YM155	Sepantronium bromide	BIRC5 (Survivin)	apoptosis regulation	11178236
YM201636		PYKfyve(FYV1)	other	9956222
Z-LLNle-CHO	Z-L-Norleucine-CHO	gamma-secretase	other	16760646
ZG-10		IRAK1	other	
ZM-447439	ZM447439	AURKB	mitosis	9914412
ZSTK474	KIN001-167	PI3K	PI3K signaling	
Zibotentan	Zibotentan	Endothelin A Receptor	other	9910224
rTRAIL		DR4, DR5	apoptosis regulation	
