# Supplementary material for: Androgen receptor as a mediator and biomarker of radioresistance in triple-negative breast cancer
Source: NPJ Breast Cancer. 2017 Aug 18;3:29. doi: 10.1038/s41523-017-0038-2 (PMC5562815; doi:10.1038/s41523-017-0038-2)
Supplement: Supplementary file 12 — Supplemental Table 3 [file 41523_2017_38_MOESM12_ESM.pdf]

**Supplementary Table 3– Servant dataset**		
**Characteristic**		**N=343 (%)** (except when data missing)
Age	≤40	78 (23%)
	>40	265 (77%)
Tumor Stage	T1	216 (63%)
	T2-3	125 (37%)
Nodal Status	Negative	215 (64%)
	Positive	128 (36%)
Recurrence Status	Local recurrence	119 (35%)
	No local recurrence	224 (65%)
Surgical Margin	Negative	318 (94%)
	Positive	22 (6%)
Boost RT (≥66 Gy)	Yes	247 (73%)
	No	97 (27%)
Adjuvant CT/HT	No	201 (59%)
	Yes	141 (41%)
Adjuvant CT	No	209 (61%)
	Yes	133 (39%)
Adjuvant HT	No	311 (91%)
	Yes	31 (9%)
Grade	I/II	202 (60%)
	III	137 (40%)
Mitotic Index	MI 1	174 (53%)
	MI 2	59 (18%)
	MI 3	96 (29%)
LVI	Absent	254 (96%)
	Present	11 (4%)
p53 Mutation	Absent	73 (75%)

	Present	35 (19%)
ER	Positive	267 (78%)
	Negative	76 (22%)
PR	Positive	204 (59%)
	Negative	139 (41%)
HER2/neu	Positive	52 (15%)
	Negative	291 (85%)
TNBC	Yes	64 (19%)
	No	279 (81%)

Supplementary Table– Clinical characteristics of the tumors in the Servant dataset. This study from a French (57%) and Dutch (43%) cohort of patients which had clinical follow-up data that allowed for the analysis of the effect of AR expression level on local recurrence. Abbreviations: RT, radiation therapy, CT, chemotherapy; HT, hormone therapy; LR, local recurrence; LVI, lobular vascular in situ; MI, mitotic index; TNBC, triple-negative breast cancer.

Supplementary Table 3– van de Vijver dataset

**Characteristic**		**N=295 (%)** (except when data missing)
Age	<40	63 (21%)
	40-49	183 (62%)
	≥ 50	49 (17%)
Tumor Stage	T1	155 (53%)
	T2-3	140 (47%)
Nodal Status	0 nodes	151 (51%)
	1-3 nodes	106 (36%)
	≥4 nodes	38 (13%)
Recurrence Status	No local recurrence	268 (91%)
	Local recurrence	27 (9%)
Adjuvant CT	No	185 (63%)
	Yes	110 (37%)

Adjuvant HT	No	255 (96%)
	Yes	40 (14%)
Grade	I/II	176 (60%)
	III	119 (40%)
LVI	Absent	185 (63%)
	Present	110 (37%)
ER	Positive	226 (77%)
	Negative	69 (23%)

Supplementary Table– Clinical characteristics of the tumors in the van de Vijver dataset. This study from a Dutch cohort of patients which had clinical follow-up data that allowed for the analysis of the effect of AR expression on local recurrence. Abbreviations: CT, chemotherapy; HT, hormone therapy; LR, local recurrence; LVI, lymphovascular invasion.
